# Supplementary material for: Quantum plasmonics pushes chiral sensing limit to single molecules: a paradigm for chiral biodetections
Source: Nat Commun. 2024 Jan 2;15:2. doi: 10.1038/s41467-023-42719-z (PMC10762144; doi:10.1038/s41467-023-42719-z)
Supplement: Supplementary file 1 — Supplementary Information [file 41467_2023_42719_MOESM1_ESM.pdf]

# Supplementary Information

# Quantum plasmonics pushes chiral sensing limit to single molecules: a paradigm for chiral biodetections

Chi Zhang[1†], Huatian Hu[2,3†], Chunmiao Ma[4†], Yawen Li[1], Xujie Wang[1], Dongyao Li[4], Artur Movsesyan[5,6], Zhiming, Wang[5], Alexander Govorov[6], Quan Gan[4]* and Tao Ding[1]*

[1] Key Laboratory of Artificial Micro/Nano Structure of Ministry of Education, School of Physics and Technology, Wuhan University, Wuhan, 430072, China

[2] Hubei Key Laboratory of Optical Information and Pattern Recognition, Wuhan Institute of Technology, Wuhan, 430205 China

[3] Center for Biomolecular Nanotechnologies, Istituto Italiano di Tecnologia, Via Barsanti 14, 73010 Arnesano, LE, Italy

[4] School of Chemistry and Chemical Engineering, Huazhong University of Science and Technology, Wuhan 430074, China

[5] Institute of Fundamental and Frontier Sciences, University of Electronic Science and Technology of China, Chengdu 610054, China

[6] Department of Physics and Astronomy, Ohio University, Athens OH 45701, USA

*Corresponding authors Email: t.ding@whu.edu.cn; ganquan@hust.edu.cn

† These authors contributed equally to this work

Table of content

**Supplementary Notes** 2

**Supplementary Figures** 24

**Supplementary Table** 30

**Supplementary References** 31

## Supplementary Notes

### 1. Synthesis of OS-1 and NMR characterizations

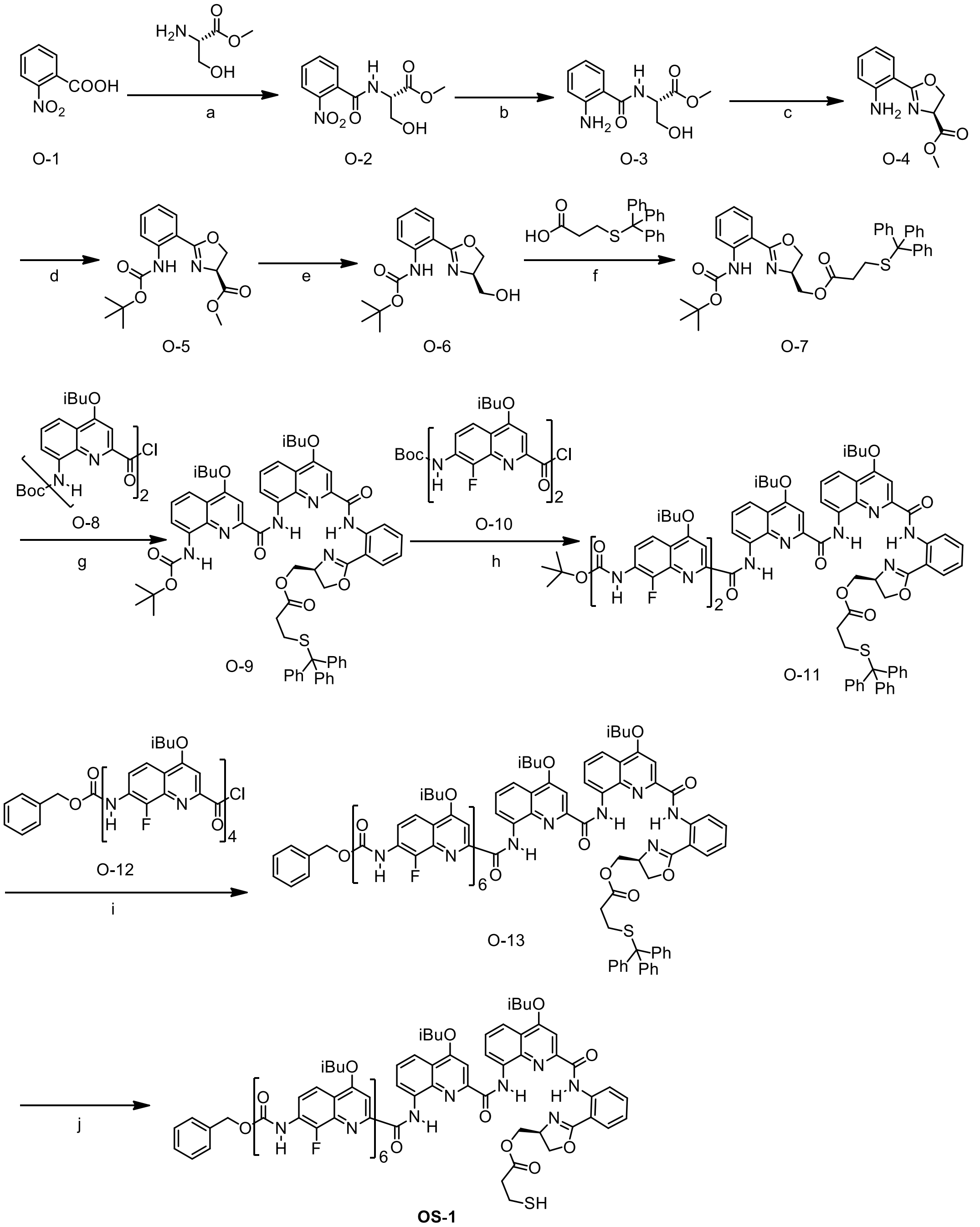


**Supplementary Fig. 1.** Synthesis of oligoamide sequences **OS-1**: **a** $(COCl)_2$, DCM, DIEA, room temperature, 5 h. **b** 10% Pd/C, $H_2$, AcOEt, $CH_3OH$, room temperature, 5 h. **c** DAST, dry DCM, room temperature, 4 h. **d** $Boc_2O$, 1,4-Dioxane, 100 °C, 12 h. **e** $NaBH_4$, dry THF, $CH_3OH$, 65 °C, 3 h. **f** DMAP, EDC, DCM, DIEA, room temperature, 5 h. **g** TFA, dry DCM, DIEA, room temperature, 12 h. **h** TFA, dry DCM, DIEA, room temperature, 12 h. **i** TFA, dry DCM, DIEA, room temperature, 12 h. **j** TFA, Triisopropylsilane, DCM, room temperature, 3 h.

**Compound O-2**. 2-Nitrobenzoic acid (400 mg, 2.39 mmol) was suspended in

anhydrous $CH_2Cl_2$ (5 mL). Oxalyl chloride (0.31 mL, 3.59 mmol) was added and the reaction was allowed to stir at room temperature for 3 h. The solvent and excess reagents were removed under vacuum and the residue was dried under vacuum for at least 2 h to yield acid chloride as a white solid. To a solution of L- or D-serine methyl ester hydrochloride (372 mg, 2.39 mmol) and distilled DIEA (0.83 mL, 0.22 mmol) in anhydrous $CH_2Cl_2$ (2 mL) was added dropwise a solution of the freshly prepared chloride in anhydrous $CH_2Cl_2$ (10 mL) via a syringe. The reaction was allowed to proceed at room temperature for 5 h. The solution was washed with $H_2O$, dried over $Na_2SO_4$, filtered, and then concentrated. The residue was purified by flash chromatography ($SiO_2$) to give compound **O-2** as a yellowish oil (0.069 g, 90% yield). $^1$H NMR (400 MHz, $CDCl_3$): δ 8.10 (d, *J* = 8.0 Hz, 1H), 7.73-7.69 (m, 1H), 7.63-7.58 (m, 2H), 6.85 (d, *J* = 6.4 Hz, 1H), 4.87-4.83 (m, 1H), 4.20-4.08 (m, 2H), 3.83 (s, 3H), 2.49 (s, 1H). $^{13}$C NMR (101 MHz, $CDCl_3$): δ 170.6, 166.7, 146.2, 133.9, 132.2, 130.7, 128.9, 124.5, 62.4, 60.5, 55.1, 52.8. ESI-HRMS: m/z calcd for $C_{11}H_{13}N_2O_6$ $[M+H]^+$ 269.0768, found 269.0811.

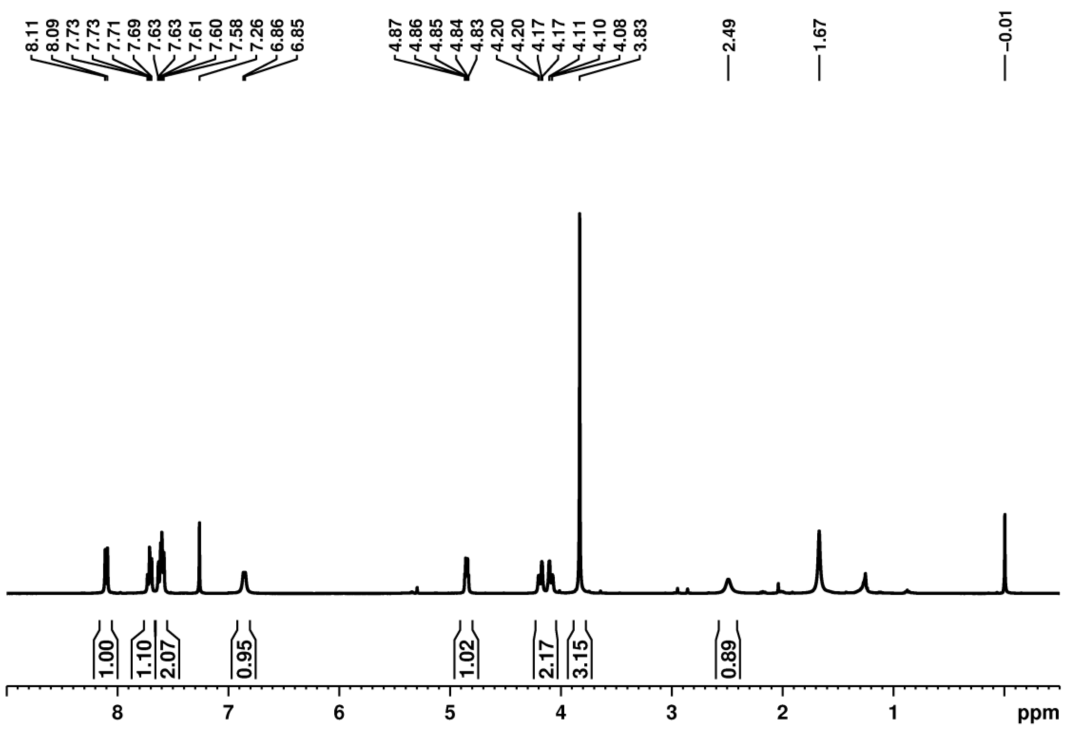


**Supplementary Fig 2.** $^1$H NMR spectrum (400 MHz) of compound **O-2** in $CDCl_3$.

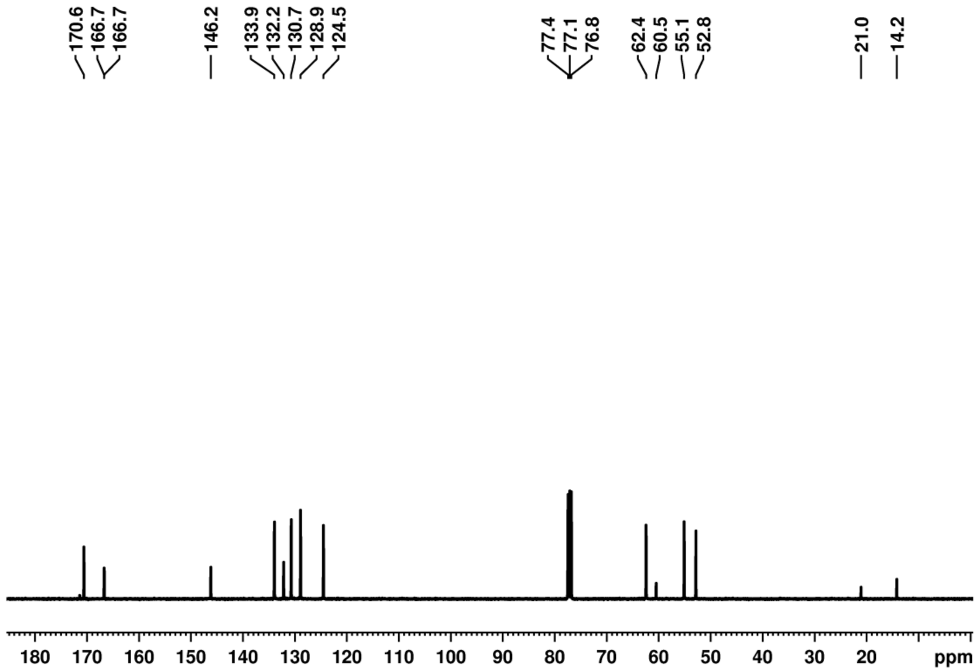


**Supplementary Fig 3.** $^{13}C$ NMR spectrum (101 MHz) of compound **O**-**2** in $CDCl_3$.

**Compound O**-**3**. To a solution of compound **O-2** (630 mg, 2.35 mmol) in AcOEt (30 mL), 10% Pd/C (63 mg) was added. The mixture was vigorously stirred at room temperature for 5 h under hydrogen atmosphere, after which complete conversion of the starting material was observed by TLC. The crude mixture was filtered through a bed of Celite and the filtrate was concentrated to dryness under reduced pressure, obtaining 500 mg (quant. yield) of compound **O-3** as a yellowish oil. $^{1}H$ NMR (400 MHz, $CDCl_3$): δ 7.46 (d, *J* = 7.8 Hz, 1H), 7.25-7.21 (m, 1H), 7.02 (d, *J* = 5.2 Hz, 1H), 6.69-6.65 (m, 2H), 5.52 (s, 1H), 4.83-4.82 (m, 1H), 4.08-4.00 (m, 2H), 3.82 (s, 3H). $^{13}C$ NMR (101 MHz, $CDCl_3$): δ 171.3, 169.5, 148.7, 132.8, 127.8, 117.4, 116.9, 115.2, 63.1, 52.8. ESI-HRMS: m/z calcd for $C_{11}H_{15}N_2O_4$ $[M+H]^+$ 239.1026, found 239.1087.

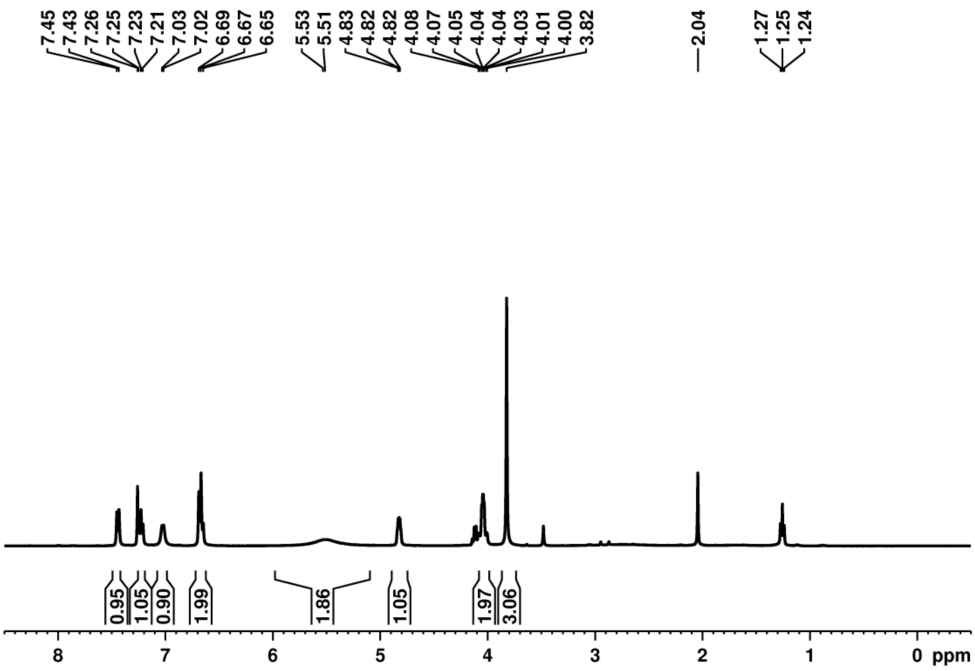


**Supplementary Fig 4.** $^{1}H$ NMR spectrum (400 MHz) of compound **O-3** in $CDCl_3$.

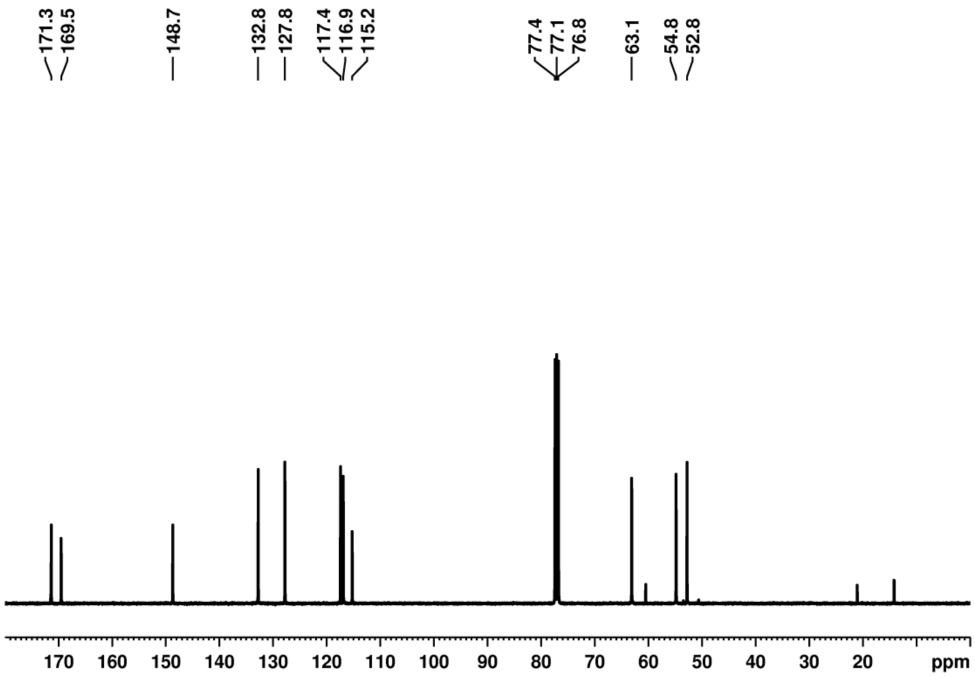


**Supplementary Fig 5.** $^{13}C$ NMR spectrum (101 MHz) of compound **O-3** in $CDCl_3$.

**Compound O-4**. To a solution of compound **O-3** (500 mg, 2.10 mmol) in dry $CH_2Cl_2$ (15 mL), DAST (0.33 mL, 2.52 mmol) was added dropwise. The mixture was stirred at room temperature for 5 h under $N_2$ atmosphere, after which complete conversion of

the starting material was observed by TLC. The solution was washed with $H_2O$, dried over $Na_2SO_4$, filtered, and then concentrated. The residue was purified by flash chromatography ($SiO_2$) to give compound **O-4** as a colorless oil (420 mg, 91% yield). $^1H$ NMR (400 MHz, $CDCl_3$): δ 7.69 (d, *J* = 7.9 Hz, 1H), 7.22 (t, *J* = 7.8 Hz, 1H), 6.69 (d, *J* = 8.2 Hz, 1H), 6.65 (t, *J* = 7.8 Hz, 1H), 6.03 (s, 1H), 5.02-4.97 (m, 1H), 4.58-4.46 (m, 2H), 3.80 (s, 3H). $^{13}C$ NMR (101 MHz, $CDCl_3$): δ 171.8, 166.5, 148.9, 132.7, 129.9, 116.0, 115.8, 108.2, 68.7, 67.9, 52.6. ESI-HRMS: m/z calcd for $C_{11}H_{13}N_2O_3$ $[M+H]^+$ 221.0921, found 221.0977.

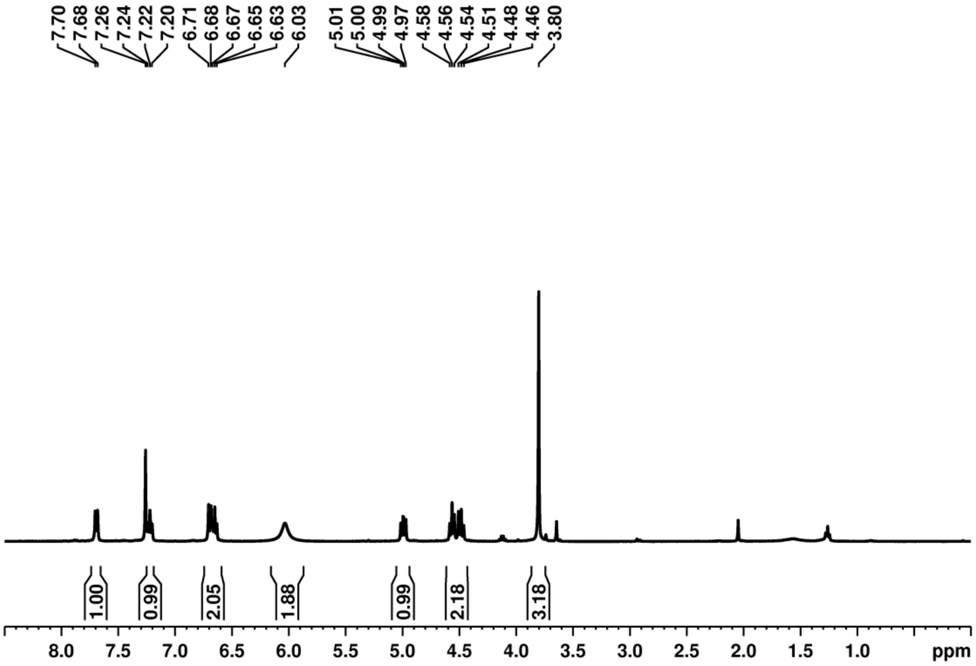


**Supplementary Fig 6.** $^1H$ NMR spectrum (400 MHz) of compound compound **O-4** in $CDCl_3$.

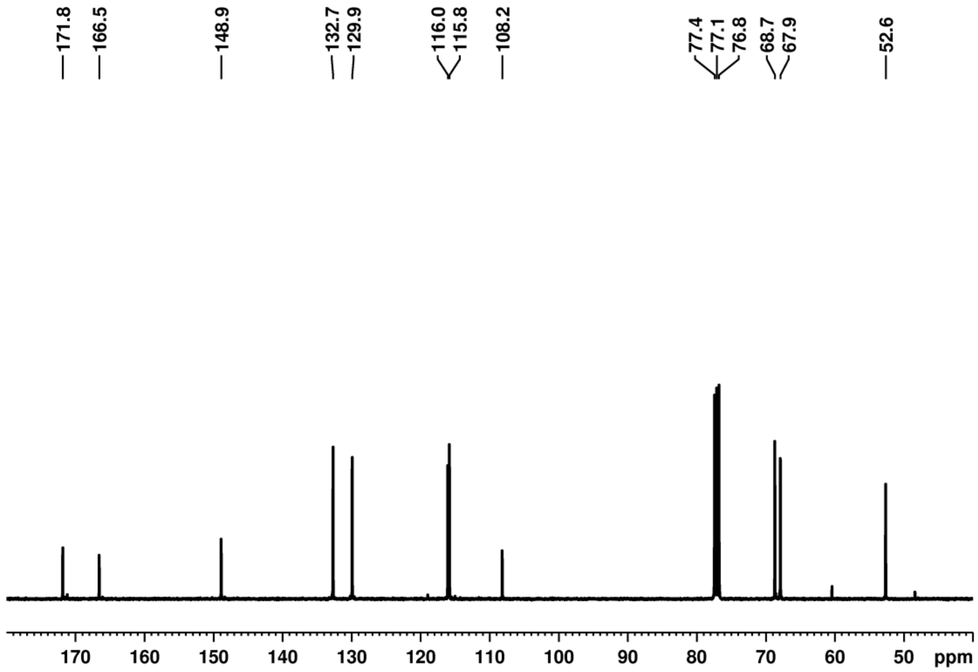


**Supplementary Fig 7.** $^{13}C$ NMR spectrum (101 MHz) of compound **O-4** in $CDCl_3$.

**Compound O-5**. To a solution of compound **O-4** (420 mg, 1.91 mmol) in dry 1,4-Dioxane (1 mL), $Boc_2O$ (0.83 mg, 3.82 mmol) was added. The mixture was stirred at 100 °C for 12 h under $N_2$ atmosphere, after which complete conversion of the starting material was observed by TLC. The residue was purified by flash chromatography ($SiO_2$) to give compound **O-5** as a colorless oil (550 mg, 90% yield). $^{1}H$ NMR (400 MHz, $CDCl_3$): δ 11.06 (s, 1H), 8.43 (d, *J* = 8.5 Hz, 1H), 7.81 (d, *J* = 7.8 Hz, 1H), 7.45 (t, *J* = 7.3 Hz, 1H), 6.99 (t, *J* = 7.4 Hz, 1H), 5.07-5.02 (m, 1H), 4.62-4.50 (m, 2H), 3.83 (s, 3H), 1.54 (s, 9H). $^{13}C$ NMR (101 MHz, $CDCl_3$): δ 171.3, 166.0, 153.2, 146.7, 140.8, 133.0, 129.5, 120.9, 118.3, 111.9, 80.1, 68.4, 68.3, 52.7, 27.4. ESI-HRMS: m/z calcd for $C_{16}H_{21}N_2O_5$ $[M+H]^+$ 321.1445, found 321.1467.

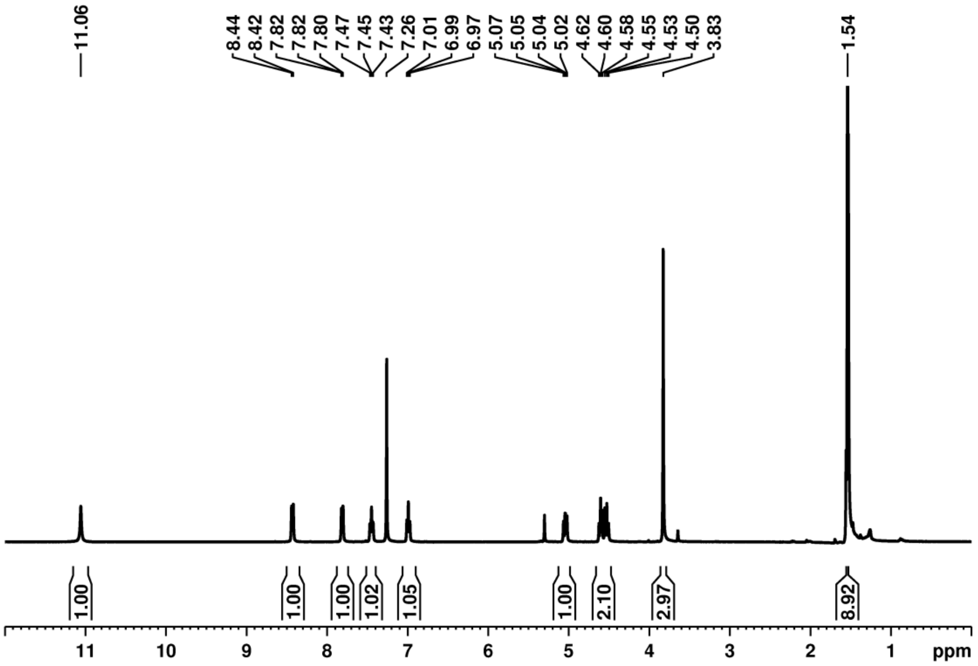


**Supplementary Fig 8.** $^{1}$H NMR spectrum (400 MHz) of compound compound **O-5** in $CDCl_3$.

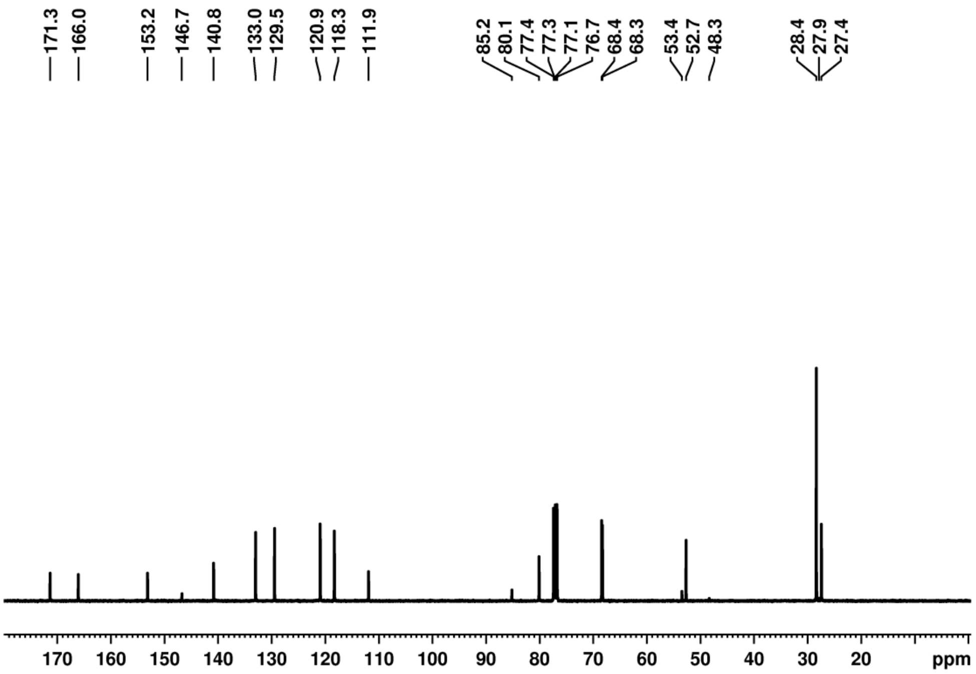


**Supplementary Fig 9.** $^{13}$C NMR spectrum (101 MHz) of compound **O-5** in $CDCl_3$.

**Compound O**-**6**. A mixture of compound **O-5** (550 mg, 1.72 mmol) and $NaBH_4$ (650 mg, 17.20 mmol) in THF (20 mL) was stirred at 65 °C for 20 min. After that, methanol (10 mL) was added dropwise during 30 min and effervescence was observed. Stirring

at 65 °C was maintained for further 2 h. The reaction was cooled to room temperature and then evaporated to generate crude solid, which was added DCM (15 mL), washed with water and brine, dried over $Na_2SO_4$. The solvents were evaporated to give crude product, which was purified by flash chromatography (silica gel) to give the desired product **O-6** (427 mg, 85% yield). $^1H$ NMR (400 MHz, $CDCl_3$): δ 11.27 (s, 1H), 8.41 (d, *J* = 8.5 Hz, 1H), 7.82 (d, *J* = 7.9 Hz, 1H), 7.44 (t, *J* = 9.4 Hz, 1H), 7.00 (t, *J* = 7.7 Hz, 1H), 4.59-4.54 (m, 1H), 4.43 (t, *J* = 8.4 Hz, 1H), 4.26 (t, *J* = 8.0 Hz, 1H), 3.94-3.89 (m, 1H), 3.73-3.67 (m, 1H), 1.53 (s, 9H). $^{13}C$ NMR (101 MHz, $CDCl_3$): δ 171.2, 165.1, 153.2, 140.6, 132.6, 129.4, 121.0, 118.1, 112.2, 80.2, 68.1, 68.0, 64.3, 60.4, 21.0, 14.2. ESI-HRMS: m/z calcd for $C_{15}H_{21}N_2O_4$ $[M+H]^+$ 293.1496, found 293.1511.

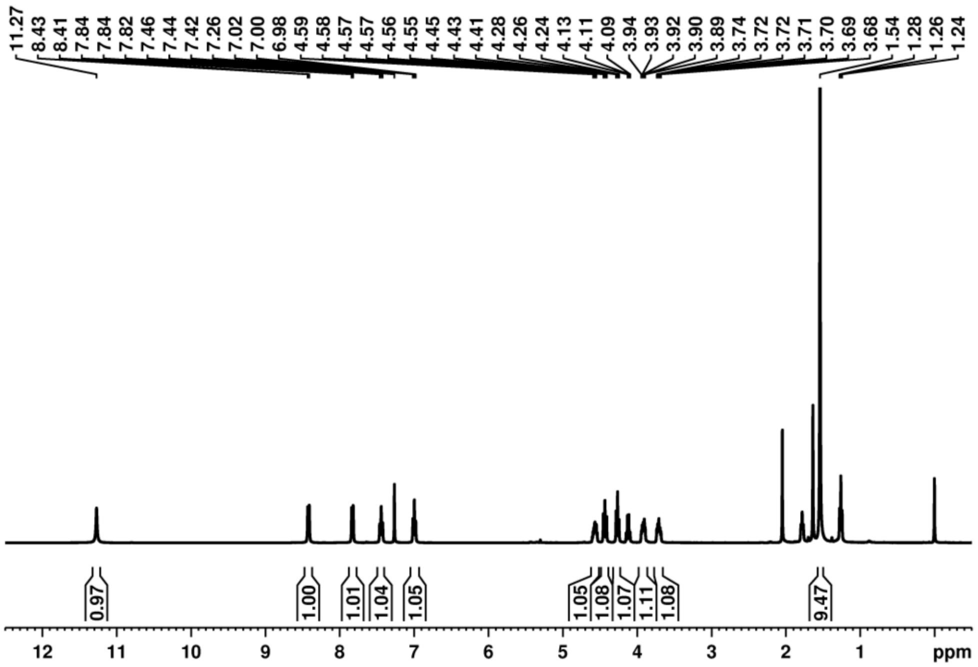


**Supplementary Fig 10.** $^1H$ NMR spectrum (400 MHz) of compound **O-6** in $CDCl_3$.

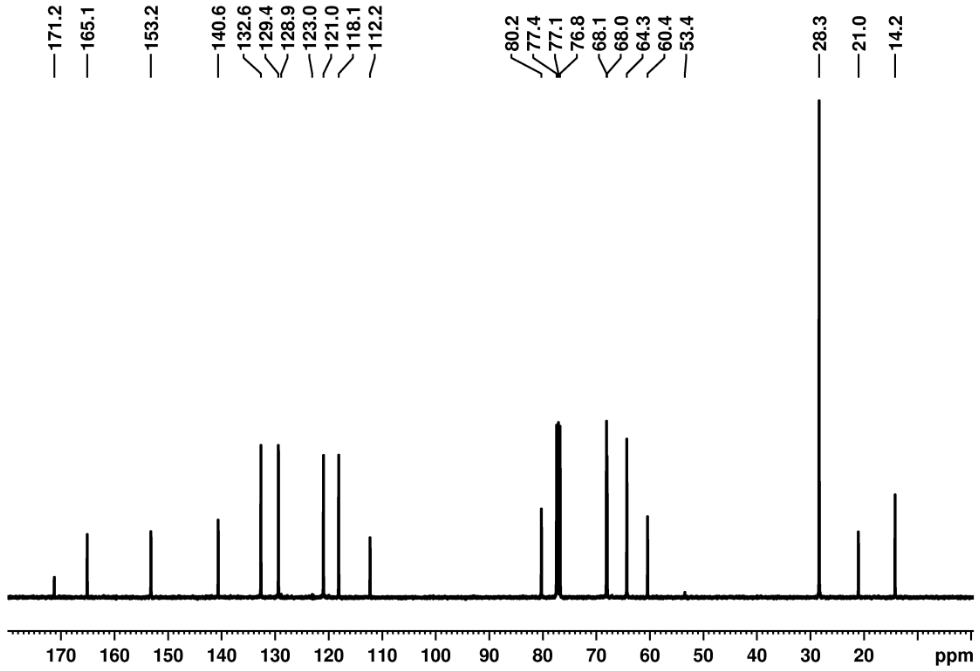


**Supplementary Fig 11.** $^{13}C$ NMR spectrum (101 MHz) of compound **O-6** in $CDCl_3$.

**Compound O-7**. Compound **O-6** (427 mg, 1.46 mmol) and 3-(tritylthio) propionic acid (560 mg, 1.61 mmol) were dissolved in $CH_2Cl_2$ (10 mL), then EDCI (426 mg, 2.19 mmol) and DMAP were added. The reaction mixture was allowed to stir at room temperature for 8 h. Then the crude solution was washed with distilled water, dried over $MgSO_4$, filtered and evaporated. The residue was purified by flash chromatography ($SiO_2$) to give compound **O-7** as a colorless oil (864 mg, 95% yield).$^{1}H$ NMR (400 MHz, $CDCl_3$): δ 11.17 (s, 1H), 8.44 (d, *J* = 8.5 Hz, 1H), 7.79 (d, *J* = 7.8 Hz, 1H), 7.46-7.39 (m, 7H), 7.29-7.25 (m, 6H), 7.22-7.18 (m, 3H), 6.98 (t, *J* = 7.7 Hz, 1H), 4.65-4.58 (m, 1H), 4.40-4.29 (m, 2H), 4.17-4.10 (m, 2H), 2.47-2.43 (m, 2H), 2.22-2.19 (m, 2H), 1.52 (s, 9H). $^{13}C$ NMR (101 MHz, $CDCl_3$): δ 171.7, 165.1, 153.2, 144.6, 140.9, 132.8, 129.9, 129.4, 128.0, 126.8, 126.4, 120.9, 118.2, 112.1, 80.2, 68.5, 66.9, 65.7, 55.2, 60.4, 53.5, 33.5, 26.8, 21.1, 14.3. ESI-HRMS: m/z calcd for $C_{37}H_{39}N_2O_5S$ $[M+H]^+$ 623.2574, found 623.2560.

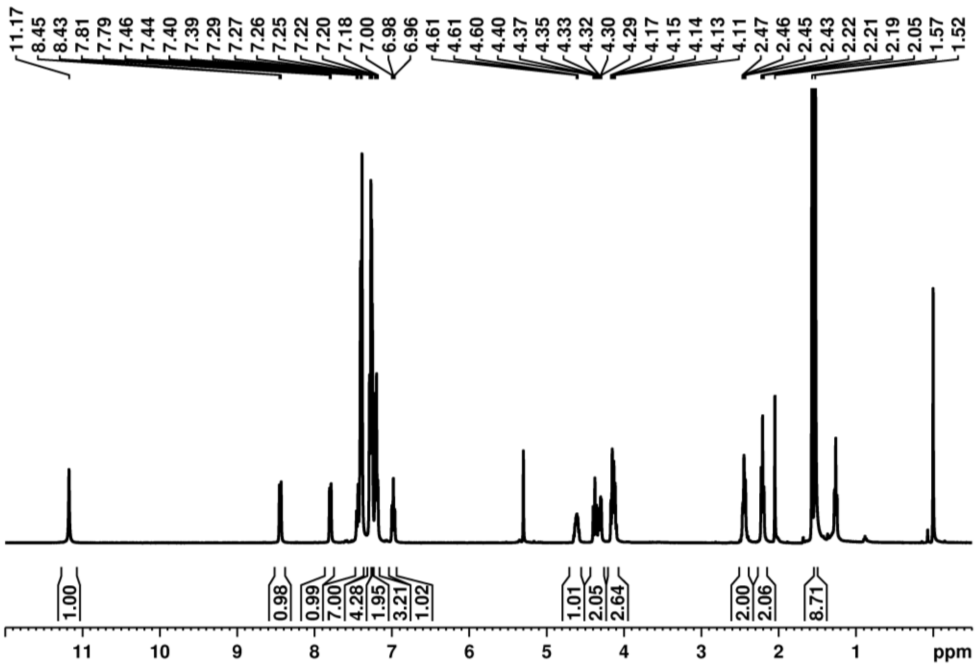


**Supplementary Fig 12.** $^{1}H$ NMR spectrum (400 MHz) of compound **O-7** in $CDCl_3$.

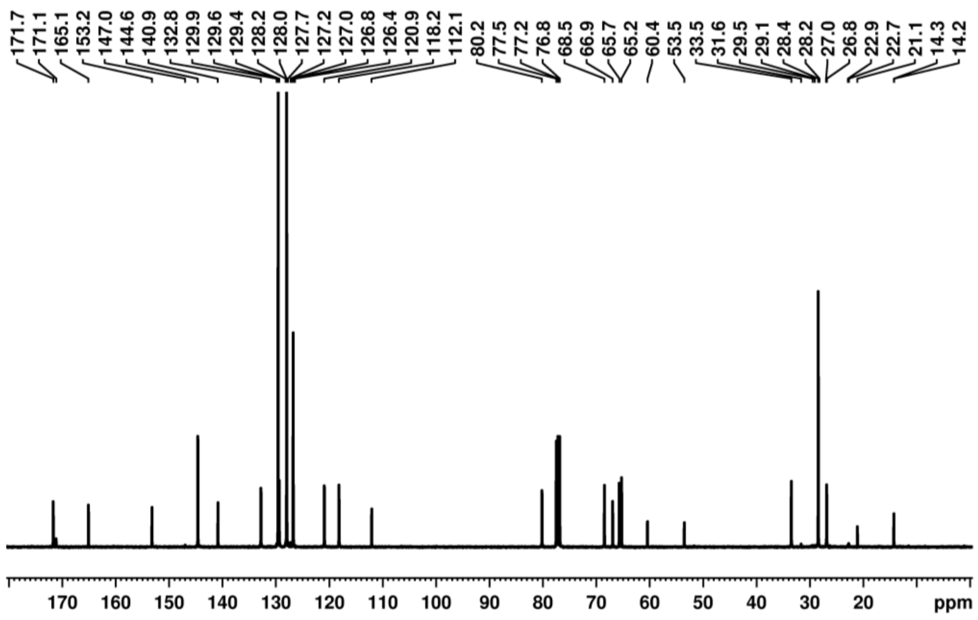


**Supplementary Fig 13.** $^{13}C$ NMR spectrum (101 MHz) of compound **O-7** in $CDCl_3$.

**Compound O-9**. TFA (4 mL) was added to a solution of compound **O-7** (864 mg, 1.39 mmol) in DCM (4 mL). The mixture was stirred at room temperature for 3 hours, after which complete conversion of the starting material was observed by TLC. Volatiles were removed in vacuo and then the residue was dissolved in $CH_2Cl_2$, washed with saturated aqueous $NaHCO_3$ and saturated aqueous NaCl, dried over

$MgSO_4$ and concentrated under reduced pressure, obtaining 800 mg (quant. yield) of amine as a yellow solid. The residue was purified by flash chromatography ($SiO_2$) and pure amine was obtained as a yellowish oil. Amine (600 mg, 1.15 mmol) and DIEA (2.0 equiv.) were dissolved in anhydrous DCM (5 mL) under argon with stirring. The acid chloride **O-8** was dissolved in anhydrous DCM (5 mL), then were added dropwise into amine liquor immediately. After stirring for overnight, TLC analysis indicated completion of the reaction. The mixture extracted with DCM. The resultant crude material was purified by column chromatography ($SiO_2$, DCM/EtOAc stepwise elution, 100:1 to 20:1) to give the desired products **O-9** (1.02 g, yield 80%). $^1$H NMR (400 MHz, $CDCl_3$): δ 13.7 (s, 1H), 12.3 (s, 1H), 9.19 (d, *J* = 8.3 Hz, 1H), 9.04 (d, *J* = 7.6 Hz, 1H), 8.64 (s, 1H), 8.03-8.01 (m, 2H), 7.85 (d, *J* = 8.3 Hz, 1H), 7.74 (d, *J* = 8.0 Hz, 2H), 7.66-7.51 (m, 4H), 7.28-7.13 (m, 16 H), 4.18-4.02 (m, 5H), 3.58-3.38 (m, 3H), 2.70-2.50 (m, 2H), 2.34-2.28 (m, 2H), 2.02-2.00 (m, 2H), 1.31 (s, 9H), 1.17-1.13 (m, 12H). $^{13}$C NMR (101 MHz, $CDCl_3$): δ 171.4, 164.5, 164.3, 163.6, 163.4, 163.1, 152.3, 151.6, 150.1, 144.6, 139.8, 138.8, 137.6, 135.3, 134.7, 132.8, 129.5, 129.4, 128.3, 127.8, 127.7, 126.6, 122.8, 122.4, 121.9, 120.2, 117.4, 116.8, 116.6, 114.2, 113.7, 99.8, 98.8, 80.6, 75.5, 75.3, 67.1, 66.6, 65.6, 64.5, 60.4, 32.8, 31.6, 29.4, 28.2, 28.1, 26.9, 26.4, 22.8, 22.7, 21.1, 19.3, 19.2, 14.2. ESI-HRMS: m/z calcd for $C_{65}H_{67}N_6O_9S$ $[M+H]^+$ 1107.4685, found 1107.4699.

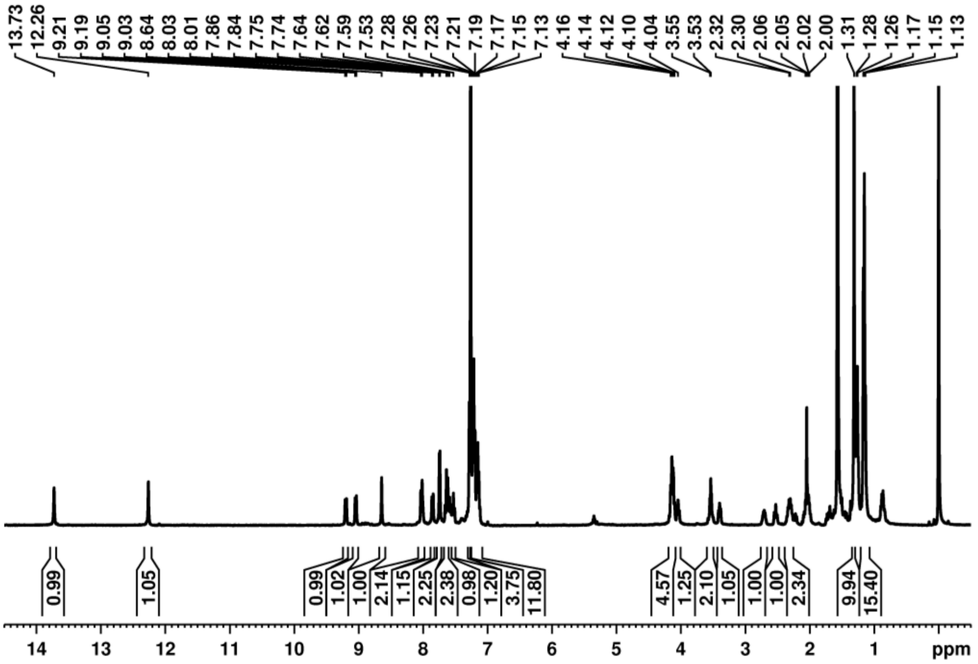


**Supplementary Fig 14.** $^1$H NMR spectrum (400 MHz) of compound **O-9** in $CDCl_3$.

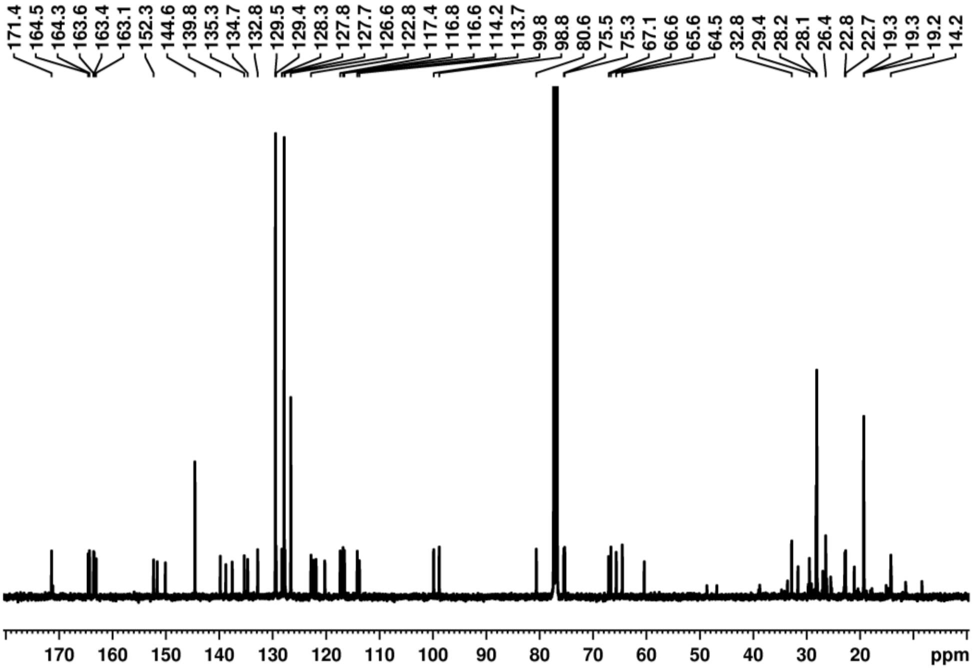


**Supplementary Fig 15.** $^{13}C$ NMR spectrum (101 MHz) of compound **O-9** in $CDCl_3$.

**Compound O-11**. This compound was prepared according to the general procedure for the synthesis of compound **O-9** from compound **O-7**. While the compound of **O-10** [2] was referred to reported papers. Yield: 1.23 g (80%). $^1H$ NMR (400 MHz, $CDCl_3$): δ 13.12 (s, 1H), 12.62 (s, 1H), 12.32 (s, 1H), 9.61 (s, 1H), 9.13 (d, *J* = 7.7 Hz, 1H), 8.78 (d, *J* = 7.3 Hz, 1H), 8.73 (t, *J* = 7.2 Hz, 1H), 8.63 (t, *J* = 7.8 Hz, 1H), 8.35 (d, *J* = 8.0 Hz, 1H), 8.09 (t, *J* = 7.9 Hz, 2H), 8.00 (d, *J* = 8.4 Hz, 1H), 7.90 (s, 1H), 7.70-7.63 (m, 3H), 7.45 (t, *J* = 7.6 Hz, 1H), 7.23-6.81 (m, 23H), 4.25-3.34 (m, 17H), 2.44-2.36 (m, 4H), 1.28-1.20 (m, 33H). $^{13}C$ NMR (101 MHz, $CDCl_3$): δ 170.3, 162.8, 162.5, 162.0, 161.7, 161.6, 161.5, 160.7, 160.5, 151.5, 150.6, 149.8, 149.6, 148.8, 143.4, 137.9, 137.8, 137.4, 133.9, 133.7, 130.9, 128.6, 128.4, 127.4, 126.7, 126.0, 125.5, 124.8, 121.8, 120.9, 120.6, 119.1, 118.8, 118.5, 118.0, 117.5, 117.0, 116.7, 116.4, 116.1, 114.9, 114.4, 112.6, 98.3, 97.5, 97.4, 96.4, 80.7, 74.4, 74.3, 74.2, 73.9, 65.8, 65.5, 64.5, 63.5, 49.8, 31.7, 28.7, 27.4, 27.2, 27.1, 25.2, 18.4, 18.3, 18.2. ESI-HRMS: m/z calcd for $C_{93}H_{93}F_2N_{10}O_{13}S$ $[M+H]^+$ 1627.6607, found 1627.6599.

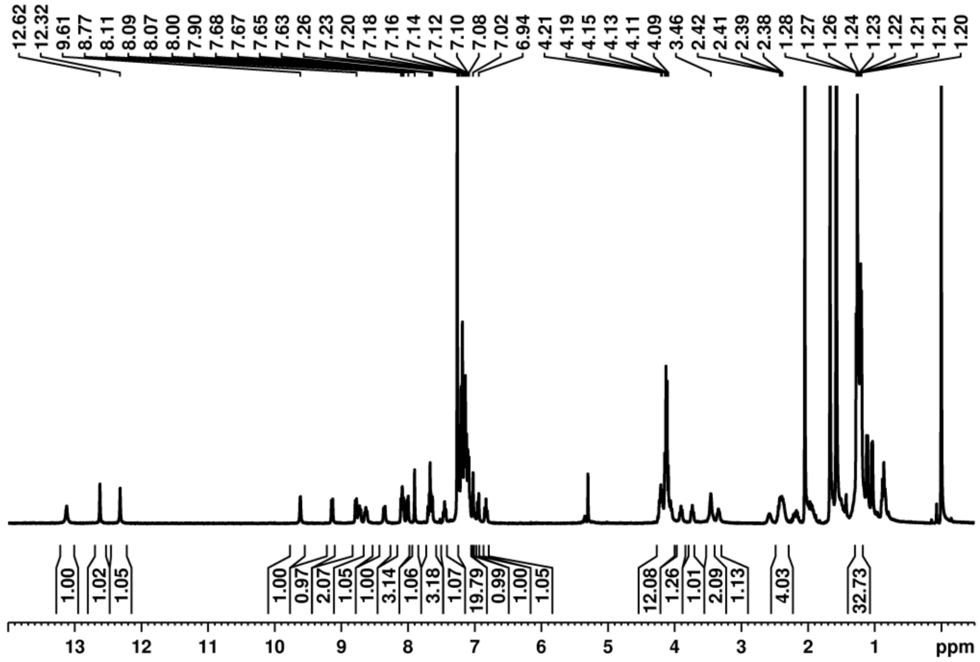


**Supplementary Fig 16.** $^1$H NMR spectrum (400 MHz) of compound **O-11** in $CDCl_3$.

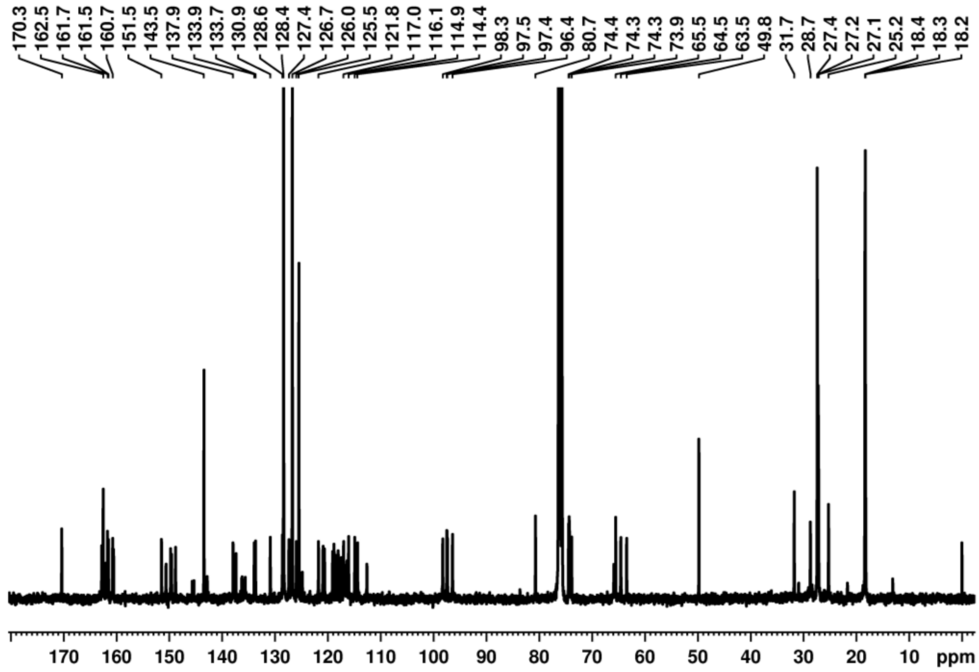


**Supplementary Fig 17.** $^{13}$C NMR spectrum (101 MHz) of compound **O-11** in $CDCl_3$.

**Compound O**-**13**. This compound was prepared according to the general procedure for the synthesis of compound **O-9** from compound **O-7**. While the compound of **O-12** *(39)* was referred to reported papers. Yield: 1.36 g (75%). $^1$H NMR (400 MHz, $CDCl_3$): δ 12.33 (s, 1H), 11.84 (s, 1H), 11.55 (s,1H), 10.85 (s, 1H), 10.11 (s, 1H), 9.76 (s,

1H), 9.49 (s, 1H), 9.41 (s,1H), 8.95 (d, *J* = 7.9 Hz, 1H), 8.74 (t, *J* = 8.0 Hz, 1H), 8.57 (t, *J* = 8.3 Hz, 1H), 8.27 (t, *J* = 8.2 Hz,1H), 8.02 (d, *J* = 7.2 Hz, 1H), 7.83-7.68 (m, 4H), 7.55 (d, *J* = 8.7 Hz, 1H), 7.52-7.44 (m, 4H), 7.18-6.67 (m, 35H), 6.62 (s, 1H), 6.38-6.24 (m, 3H), 4.98 (d, *J* = 12.2 Hz, 1H), 4.17-2.82 (m, 24H), 2.43-2.00 (m, 8H), 1.32-1.16 (m, 48H). $^{13}$C NMR (101 MHz, $CDCl_3$): δ 171.0, 163.0, 162.2, 162.0, 162.1, 161.9, 161.6, 161.5, 161.4, 161.3, 160.9, 160.6, 160.5, 160.4, 152.6, 150.2, 149.9, 149.7, 149.5, 149.1, 147.3, 146.9, 146.4, 145.7, 144.7, 144.3, 143.8, 143.3, 143.2, 142.9, 139.0, 138.4, 137.3, 136.7, 135.9, 135.4, 134.9, 133.9, 131.2, 129.2, 127.8, 127.5, 127.1, 126.5, 126.3, 125.9, 125.2, 122.1, 121.3, 120.4, 119.6, 118.9, 118.7, 118.4, 118.2, 118.1, 117.8, 117.7, 117.3, 116.9, 116.8, 116.5, 116.1, 115.8, 115.3, 114.0, 113.1, 98.4, 97.9, 97.2, 97.1, 96.9, 96.7, 96.4, 75.3, 74.9, 74.7, 74.6, 74.3, 74.0, 66.4, 66.3, 64.9, 63.9, 50.8, 32.4, 29.7, 29.4, 28.4, 28.2, 27.8, 26.0, 19.5, 19.4, 19.3, 19.1, 18.9, 18.8. ESI-HRMS: m/z calcd for $C_{152}H_{143}F_6N_{18}O_{21}S$ $[M+H]^+$ 2702.0295, found 2702.0223.

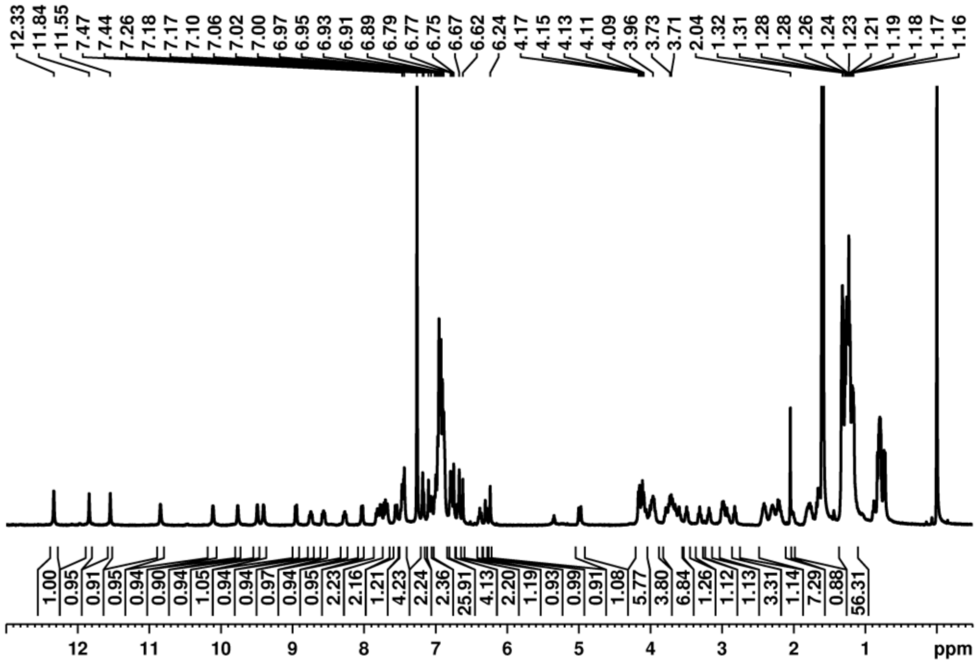


**Supplementary Fig 18.** $^1$H NMR spectrum (400 MHz) of compound **O-13** in $CDCl_3$.

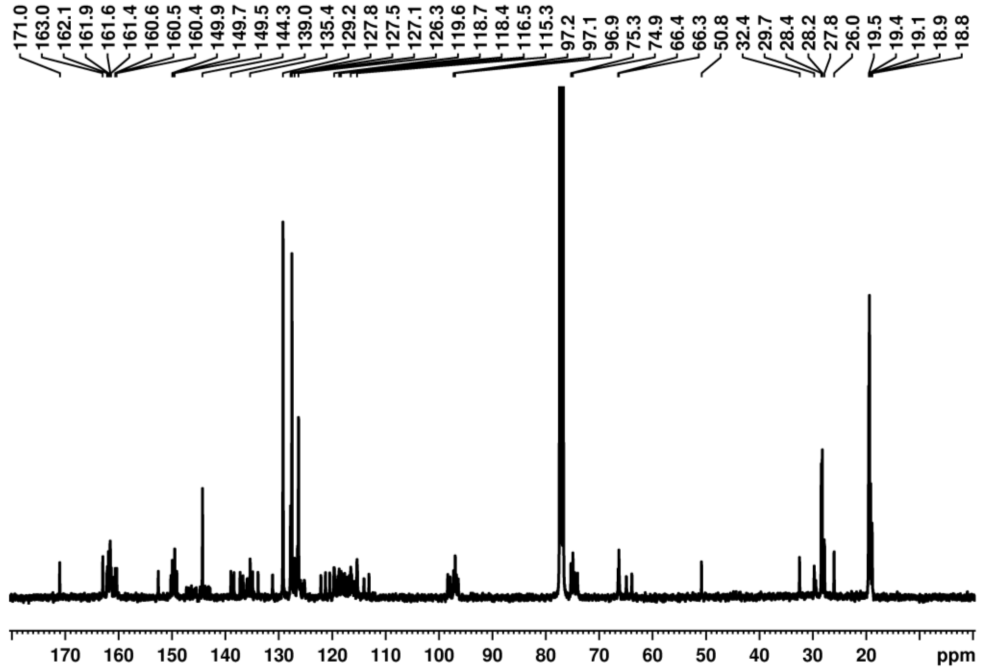


**Supplementary Fig 19.** $^{13}C$ NMR spectrum (101 MHz) of compound **O-13** in $CDCl_3$.

**Compound OS-1**. Under $N_2$ atmosphere, compound **O-13** (200 mg, 0.07 mmol) were dissolved in DCM: TFA (4 mL, 4 mL), and triisopropylsilane (0.10 mL, 0.49 mmol) was added to produce a yellow solution. The reaction mixture was stirred for 2.5 h. Volatiles were removed in vacuo and then the residue was dissolved in $CH_2Cl_2$, washed with saturated aqueous $NaHCO_3$ and saturated aqueous NaCl, dried over $MgSO_4$ and concentrated under reduced pressure. The residue was purified by flash chromatography ($SiO_2$) and pure amine was obtained as a white solid (160 mg, 88%). $^1H$ NMR (400 MHz, $CDCl_3$): δ 12.36 (s, 1H), 11.86 (s, 1H), 11.57 (s, 1H), 10.86 (s, 1H), 10.13 (s, 1H), 9.72 (s, 1H), 9.49 (s, 1H), 9.43 (s, 1H), 8.98 (d, *J* = 7.9 Hz, 1H), 8.71 (t, *J* = 8.3 Hz, 1H), 8.55 (t, *J* = 8.3 Hz, 1H), 8.26 (t, *J* = 8.2 Hz,1H), 8.01 (d, *J* = 7.2 Hz, 1H), 7.83-7.68 (m, 3H), 7.55-7.44 (m, 6H), 7.31-6.65 (m, 25H), 6.43-6.22 (m, 3H), 4.99 (d, *J* = 12.2 Hz, 1H), 4.17-2.83 (m, 20H), 2.45-1.81 (m, 12H), 1.33-1.16 (m, 48H). $^{13}C$ NMR (101 MHz, $CDCl_3$): δ 170.6, 163.0, 162.9, 162.2, 162.1, 161.9, 161.8, 161.6, 161.5, 161.4, 160.9, 160.6, 160.5, 160.3, 152.5, 150.2, 149.9, 149.7, 149.5, 149.2, 139.0, 138.4, 137.3, 136.7, 136.6, 136.0, 135.8, 135.7, 135.4, 135.0, 133.9, 131.2, 129.2, 127.8, 127.2, 127.0, 126.5, 126.4, 126.0, 125.4, 125.3, 125.2, 125.1, 122.1, 121.3, 120.4, 119.7, 119.5, 118.9, 118.6, 118.3, 118.2, 118.0, 117.7, 117.3, 117.0, 116.9, 116.4, 116.3, 116.1, 115.8, 115.3, 114.0, 113.1, 98.4, 97.9, 97.2, 97.1, 96.9, 96.4, 75.3, 74.9, 74.7, 74.5, 74.3, 74.0, 66.5, 66.3, 65.3, 63.9, 37.4, 28.4, 28.2, 27.9, 27.8, 19.5, 19.4, 19.3, 19.1, 18.9, 18.8, 18.7. ESI-HRMS: m/z calcd for $C_{133}H_{129}F_6N_{18}O_{21}S$ $[M+H]^+$ 2459.9199, found 2459.9120.

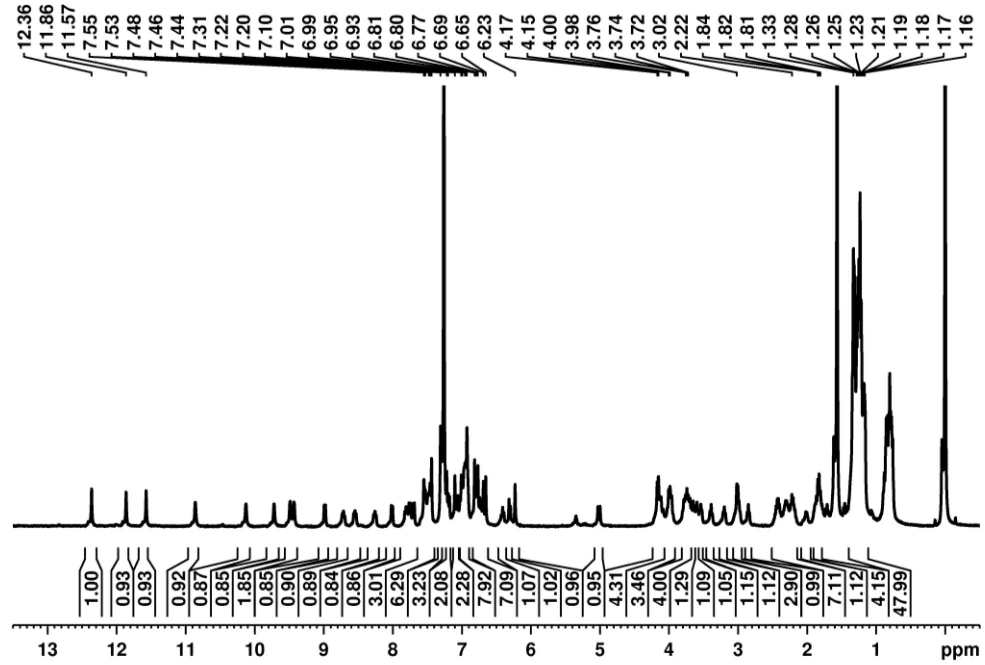


**Supplementary Fig 20.** $^{1}$H NMR spectrum (400 MHz) of compound **OS-1** in $CDCl_3$.

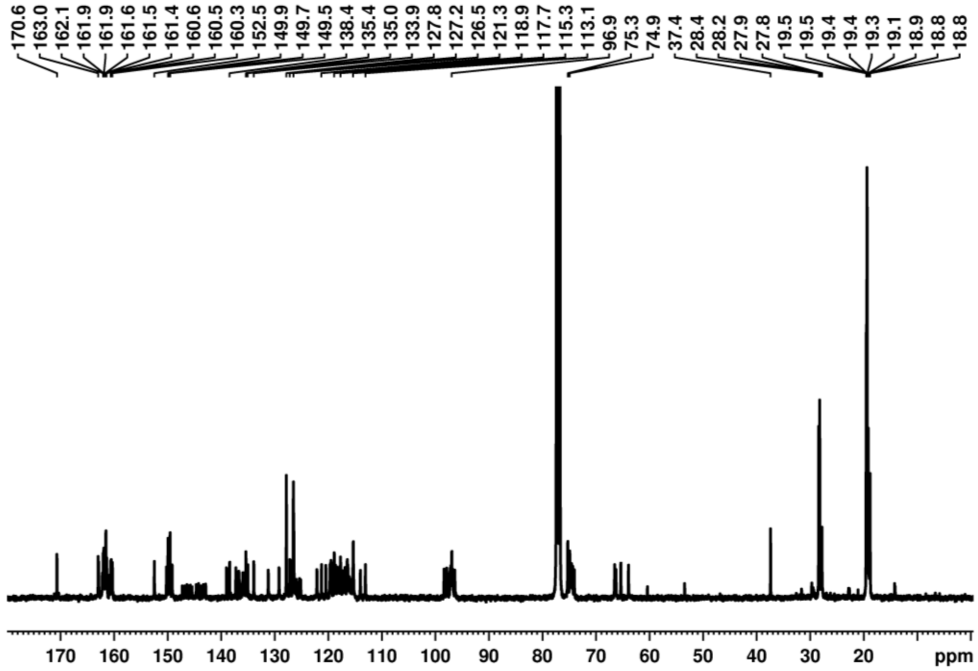


**Supplementary Fig 21.** $^{13}$C NMR spectrum (101 MHz) of compound **OS-1** in $CDCl_3$.

**Solution studies of chiral oligoamide foldamers**

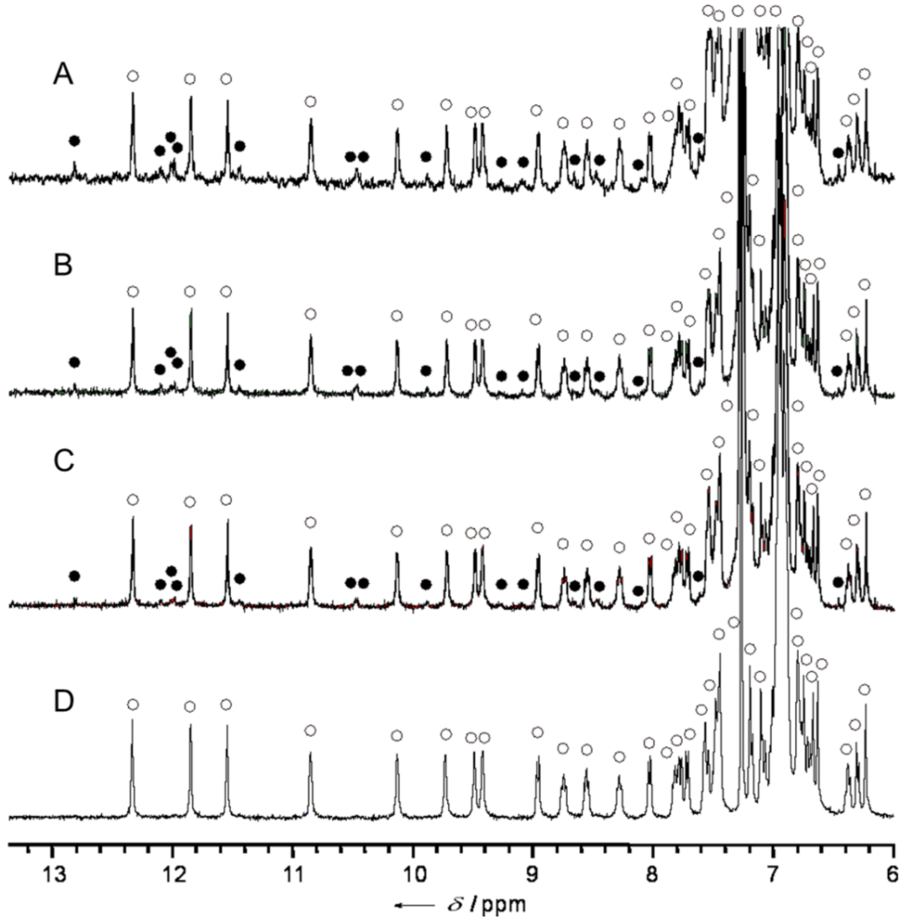


**Supplementary Fig 22.** Part of 400 MHz $^1$H NMR of compound **OS-1** in $CDCl_3$ at 296 K in various concentration: **a** 0.1 mM, **b** 0.5 mM, **c** 1 mM, **d** 5 mM. *K*a = $1.1\times10^5$ L· $mol^{-1}$.

## 2. Synthesis of OS-2 and NMR characterizations

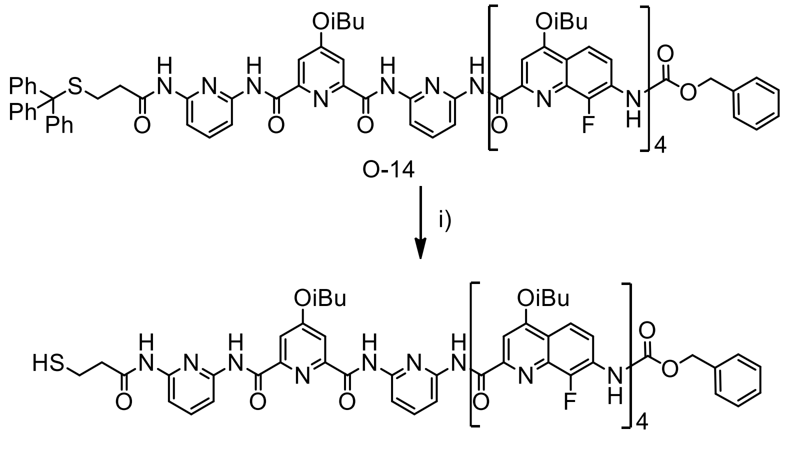


**OS-2**

**Supplementary Fig. 23. Synthesis of OS-2**: (i) Trifluoroacetic acid (TFA), Triisopropylsilane, DCM, room temperature, 3 h.

**OS-2**. Under $N_2$ atmosphere, **O-14** [1] (300 mg, 0.16 mmol) were dissolved in DCM: TFA (3:1, 4 mL), and triisopropylsilane (0.16 mL, 0.78 mmol) was added to produce a yellow solution. The reaction mixture was stirred for 2.5 h, then the crude was neutralized with a saturated aqueous solution of $NaHCO_3$, extracted with DCM (3×50 mL). The solvent was removed under reduced pressure, and the crude was washed with hexane to give the product as a white solid (240 mg, 91% yield) which was operated without further purification. $^1$H NMR (600 MHz, $CDCl_3$): δ 10.55 (s, 1H), 10.48 (s, 1H), 10.31 (s, 1H), 10.05 (s, 1H), 9.80 (s, 1H), 9.59 (s, 1H), 9.57 (s, 1H), 8.82 (t, 1H), 8.55 (t, 1H), 8.48 (t, 1H), 7.93 (d, *J* = 8.6 Hz, 1H), 7.72 (d, *J* = 7.5 Hz, 1H), 7.66(s, 1H), 7.64 (s, 1H), 7.52 (s, 1H), 7.44–7.32 (m, 4H), 7.27–7.17 (m, 4H), 7.13 (t, 1H), 7.08 (d, 1H), 6.93–6.78 (m, 8H), 4.87 (d, *J* = 12.5 Hz, 1H), 4.25–4.17 (m, 4H), 4.16–4.09 (m, 4H), 4.06 (t, 1H), 4.00–3.94 (m, 2H), 3.66 (t, 1H), 3.50 (t, 1H), 2.50–2.38 (m, 5H), 2.34 (m, 2H), 2.25 (t, 1H), 1.29–1.21 (m, 18H), 1.09 (d, *J* = 6.5 Hz, 12H). $^{13}$C NMR (101 MHz, $CDCl_3$): δ 168.3, 166.2, 161.8, 161.5, 160.8, 160.6, 160.5, 160.3, 160.1, 159.8, 159.5, 151.1, 149.8, 149.6, 149.1, 148.6, 148.5, 148.2, 147.9, 147.5, 147.3, 145.9, 145.6, 145.4, 145.0, 143.4, 143.1, 142.8, 142.5, 138.6, 138.4, 138.1, 135.7, 135.3, 135.3, 133.9, 126.9, 126.6, 126.2, 125.6, 125.3, 118.4, 118.4, 117.9, 117.7, 117.4, 117.3, 117.1, 116.4, 116.1, 115.6, 110.4, 110.2, 109.3, 108.6, 108.1, 96.6, 96.0, 95.7, 74.5, 74.3, 73.9, 73.8, 65.6, 38.8, 27.3, 27.0, 18.3, 18.1. ESI-HRMS: m/z calcd for $C_{88}H_{86}F_4N_{15}O_{14}S$ $[M+H]^+$ 1684.6130, found 1684.6136.

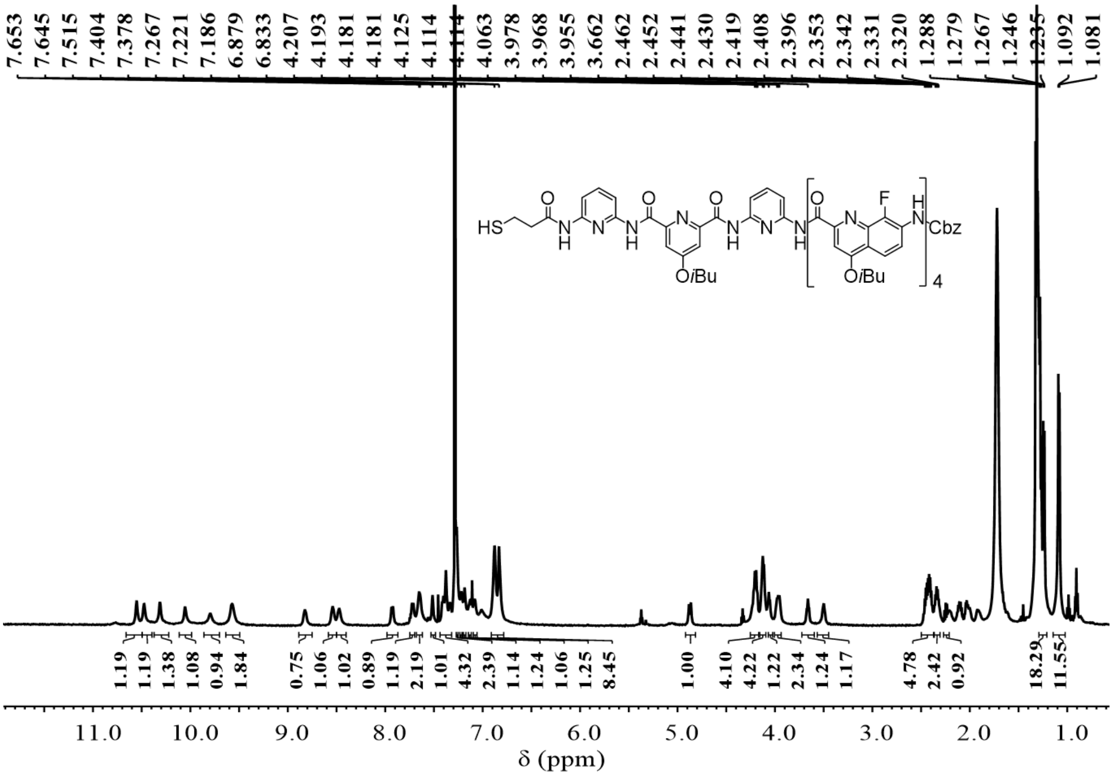


**Supplementary Fig. 24.** $^1$H NMR spectrum (400 MHz) of **OS-2** in $CDCl_3$.

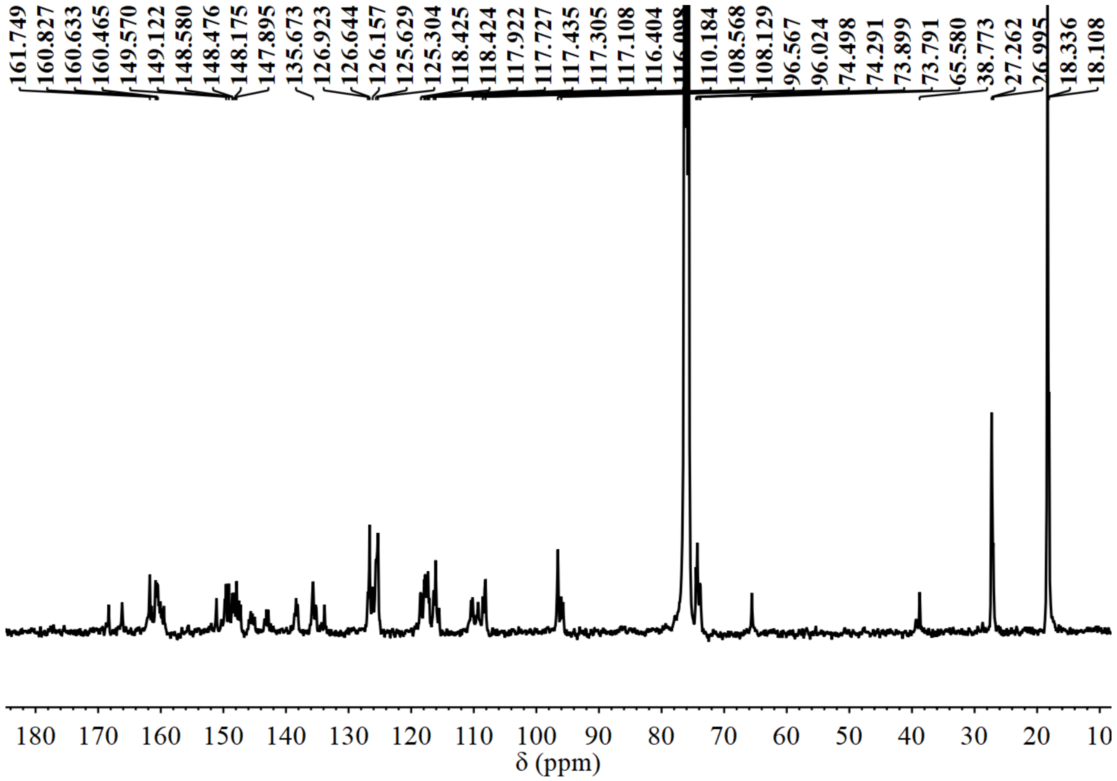


**Supplementary Fig. 25.** $^{13}C$ NMR spectrum (101 MHz) of **OS-2** in $CDCl_3$.

## 3. Synthesis of OS-3 and NMR characterizations

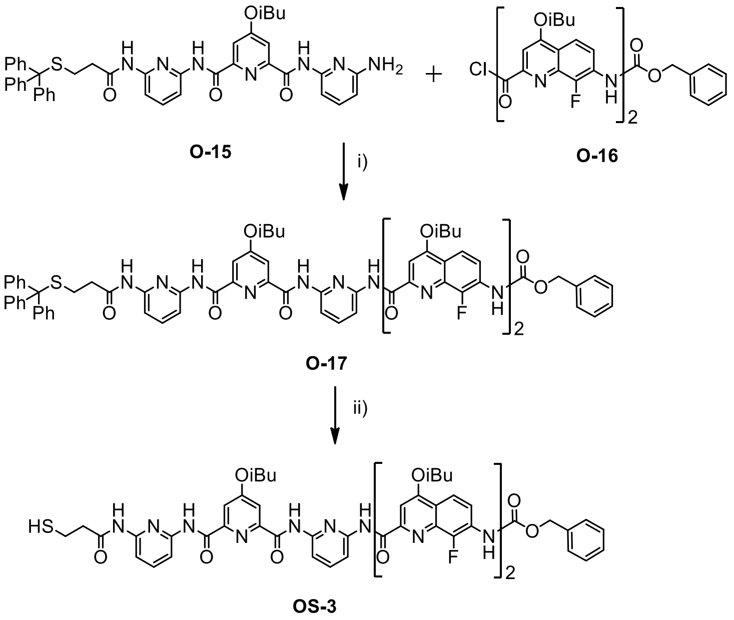


**Supplementary Fig. 26. Synthesis of OS-3**: DIEA, DCM, room temperature, 12 h.

**O-17**. A solution of the acid chloride **O-15** [1] (375 mg, 0.5 mmol) in 4 mL dry DCM was added to a solution of **O-16** [1] (350 mg, 0.5 mmol) and DIEA (0.25 mL, 1.5 mmol) in 2

mL dry DCM. The reaction was allowed to proceed overnight. The solvent was removed under reduced pressure. The crude was purified by flash column chromatography ($SiO_2$) eluting with EtOAc/DCM (1:99 to 50:100) to give the product as a white solid (0.41 g, 58% yield). $^1$H NMR (400 MHz, $CDCl_3$): δ 10.67 (s, 1H), 10.45 (s, 1H), 10.32 (s, 1H), 10.04 (s, 1H), 8.65 (s, 1H), 8.35 (s, 1H), 7.95 (d, *J* = 9.2 Hz, 1H), 7.80 (m, 6H), 7.60–7.31 (m, 10H), 7.16 (s, 1H), 7.12–6.89 (m, 15H), 5.24 (s, 2H), 4.09 (d, *J* = 6.0 Hz, 2H), 3.96 (s, 2H), 3.85 (s, 2H), 2.45–2.05 (m, 3H), 2.05 (s, 2H), 1.26 (s, 2H), 1.18 (t, *J* = 6.1 Hz, 12H), 1.10 (d, *J* = 6.7 Hz, 6H). $^{13}$C NMR (101 MHz, $CDCl_3$): δ 169.8, 167.8, 163.1, 162.9, 162.4, 161.7, 161.5, 161.1, 153.1, 151.3, 150.63 , 150.2, 149.9, 149.5, 149.3, 149.2, 149.0, 144.4, 140.2, 137.0, 136.0, 129.3, 128.7, 128.3, 127.6, 126.9, 126.3, 120.5, 119.6, 119.0, 118.9, 117.5, 117.4, 112.1, 111.3, 110.5, 109.7, 109.5, 98.1, 97.8, 75.4, 75.2, 67.2, 66.3, 35.2, 28.2, 28.1, 26.5, 19.3, 19.1. ESI-HRMS: m/z calcd for $C_{79}H_{74}F_2N_{11}O_{10}S$ $[M+H]^+$ 1406.5303, found 1406.5309.

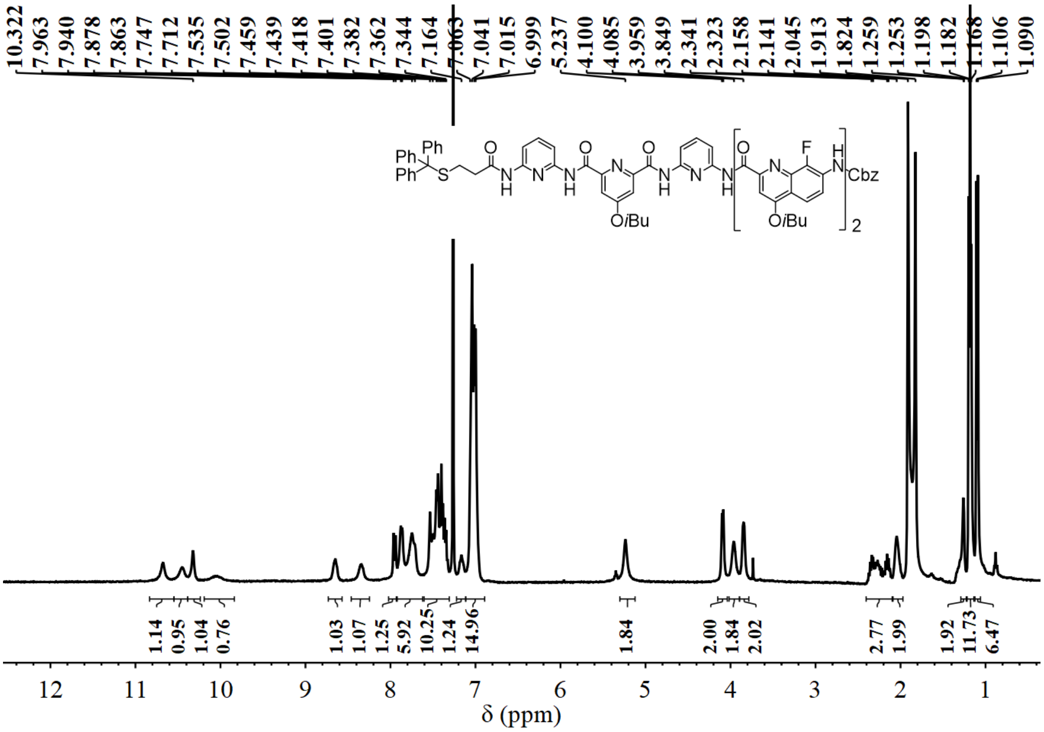


**Supplementary Fig. 27.** $^1$H NMR spectrum (400 MHz) of **O-18** in $CDCl_3$.

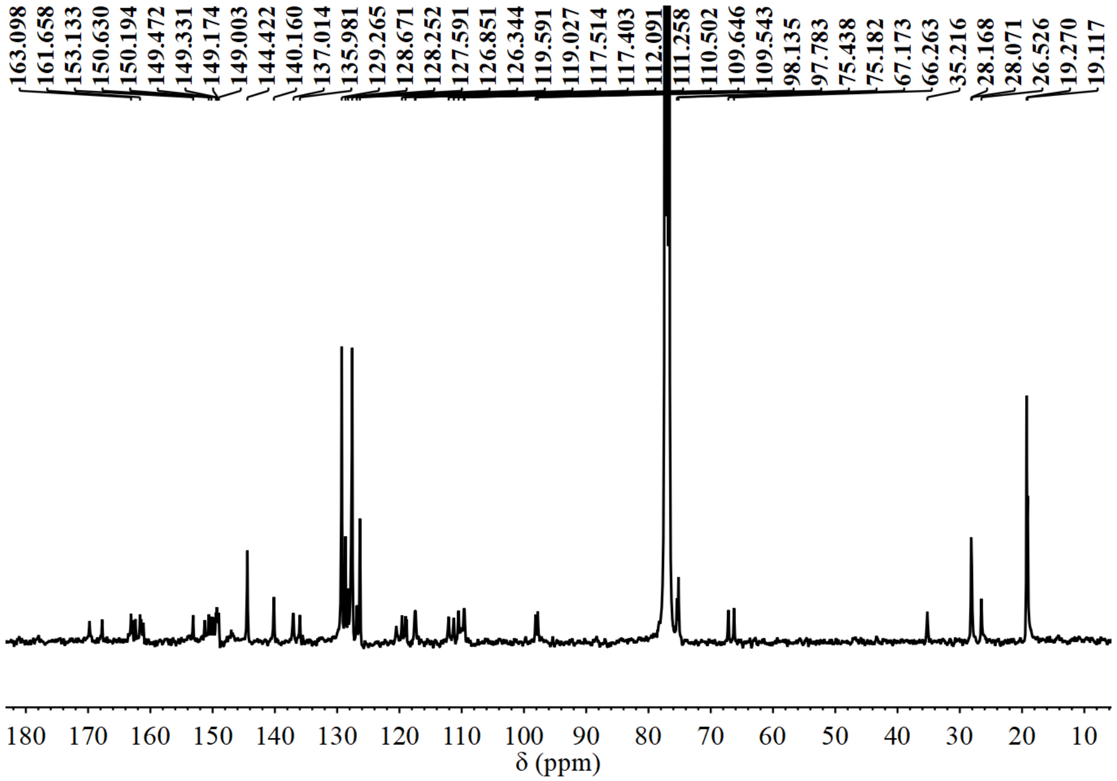


**Supplementary Fig. 28.** $^{13}C$ NMR spectrum (101 MHz) of **O-18** in $CDCl_3$.

**OS-3**. Under $N_2$ atmosphere, **O-17** (300 mg, 0.16 mmol) were dissolved in DCM: TFA (3:1, 4 mL), and triisopropylsilane (0.22 mL, 1.05 mmol) was added to produce a yellow solution. The reaction mixture was stirred for 2.5 h, then the crude was neutralized with a saturated aqueous solution of $NaHCO_3$, extracted with DCM (3×50 mL). The organic solvent was removed under reduced pressure, and the crude was washed with hexane to give the product as a white solid (230 mg, 91% yield) which was operated without further purification. $^{1}H$ NMR (400 MHz, $CDCl_3$): δ 10.62 (s, 1H), 10.44 (s, 1H), 10.41 (s, 1H), 10.19 (s, 1H), 9.33 (s, 1H), 8.68 (t, 1H), 8.37 (t, 1H), 7.98–7.88 (m, 2H), 7.88–7.75 (m, 3H), 7.71 (d, *J* = 12.6 Hz, 2H), 7.62 (t, 1H), 7.51 (s, 1H), 7.38 (m, 7H), 7.18 (s, 1H), 5.20 (s, 2H), 4.11 (d, *J* = 6.5 Hz, 2H), 4.06 (d, 2H), 3.87 (d, *J* = 6.5 Hz, 2H), 2.45–2.26 (m, 4H), 2.18 (m, 3H), 1.25 (s, 1H), 1.20 (d, *J* = 6.7 Hz, 12H), 1.12 (d, *J* = 6.7 Hz, 6H). $^{13}C$ NMR (101 MHz, $CDCl_3$): δ 170.0, 167.9, 163.1, 162.8, 162.2, 161.6, 161.2, 153.0, 151.0, 150.5, 150.3, 149.7, 149.2, 149.1, 146.9, 146.5, 144.4, 144.0, 140.6, 136.9, 135.8, 128.6, 128.2, 128.0, 126.8, 120.2, 119.5, 119.1, 118.7, 117.6, 117.4, 112.0 , 111.4, 110.3, 110.1, 109.8, 98.0, 97.7, 75.4, 75.3, 67.3, 40.2, 28.2, 28.1, 19.3, 19.1. ESI-HRMS: m/z calcd for $C_{60}H_{60}F_2N_{11}O_{10}S$ $[M+H]^+$ 1164.4208, found 1164.4215.

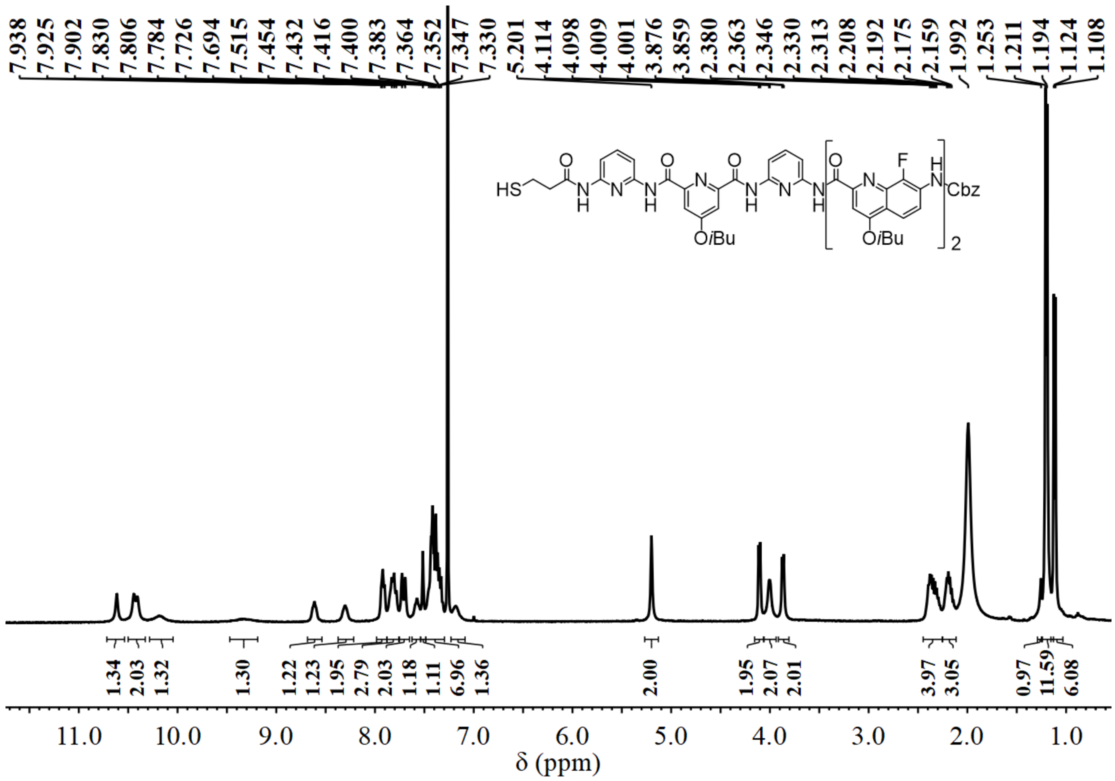


**Supplementary Fig. 29.** $^{1}H$ NMR spectrum (400 MHz) of **OS-3** in $CDCl_3$.

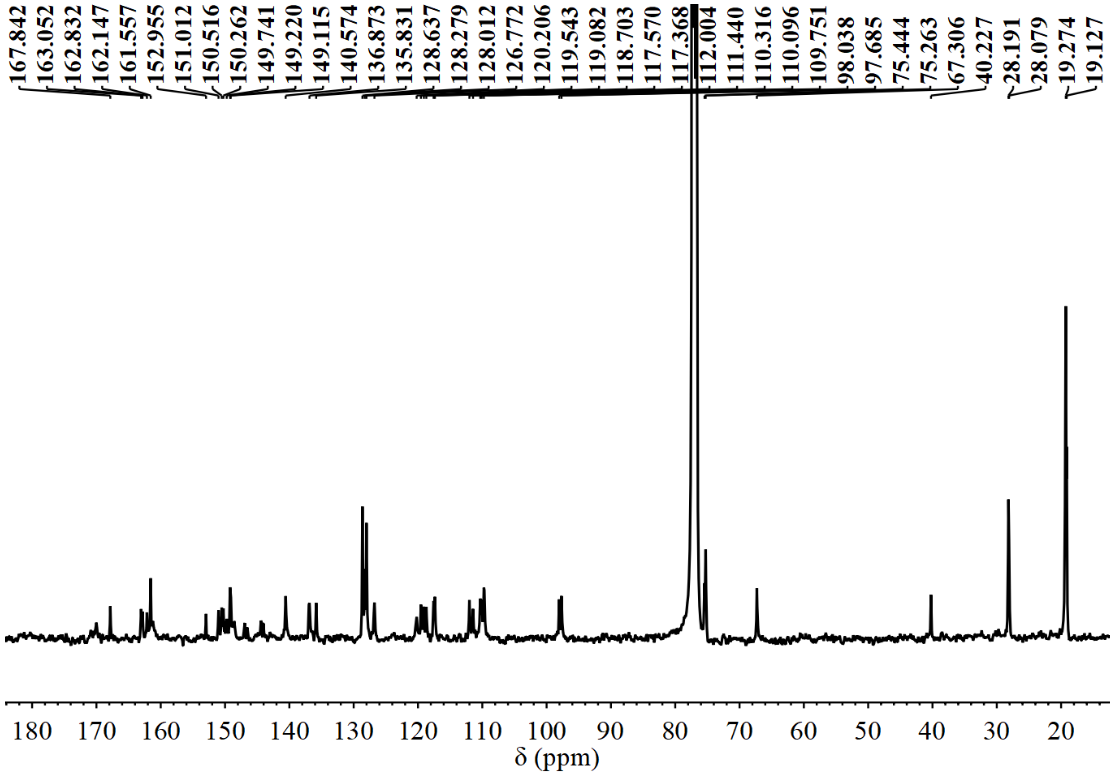


**Supplementary Fig. 30.** $^{13}C$ NMR spectrum (101 MHz) of **OS-3** in $CDCl_3$.

## Supplementary figures

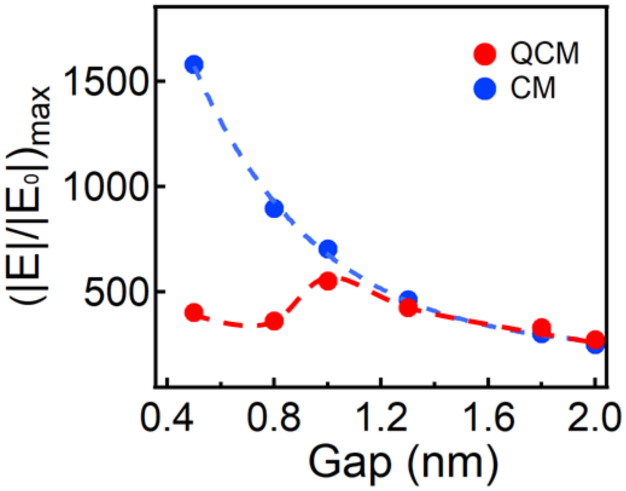


**Supplementary Fig. 31. Calculated E-field intensity of Au NPoM with different gap sizes.** Blue dash line is based on classical model (CM) and red dash line is based on quantum corrected model (QCM).

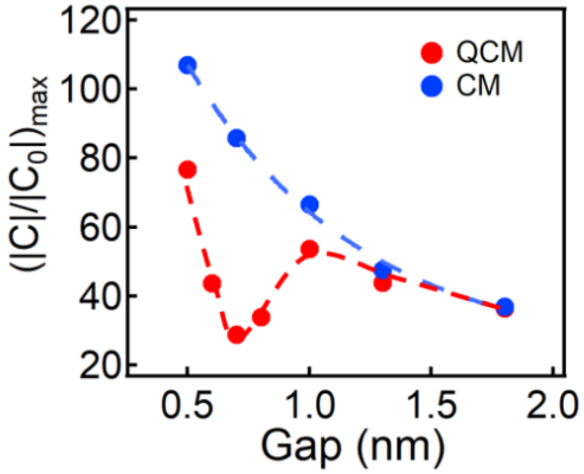


**Supplementary Fig. 32. Calculated maximum optical chirality (|C|/|C0|) of Au NPoM at different gap sizes.** Blue dash line is based on classical model (CM) and red dash line is based on quantum corrected model (QCM).

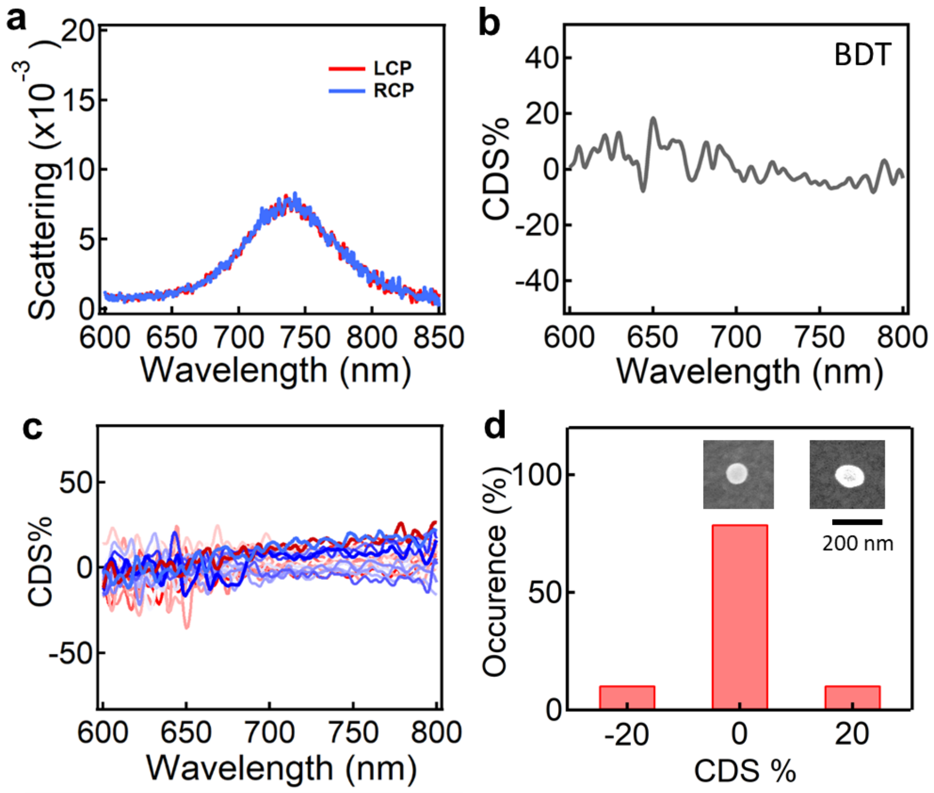


**Supplementary Fig. 33. CDS response of Au NPoM with achiral molecules (1,4-Benzenedithiol, BDT) in the nanogap. a** Scattering spectra of Au NPoM/BDT with LCP and RCP incidences and **b** the corresponding CDS spectrum. **c** Randomly collected CDS spectra of Au NPoM with BDT in the nanogap. **d** Statistics on the CDS intensity of Au NPoM/BDT based on 20 individual NPs. Inset are the representative SEM images of Au NPoMs with CDS intensity of 0 and 20% respectively. Clearly, the minor portion that found to have 20% of CDS intensity are mainly due to the nonspherical-shaped Au NPs which leads to strong extrinsic structural chirality.

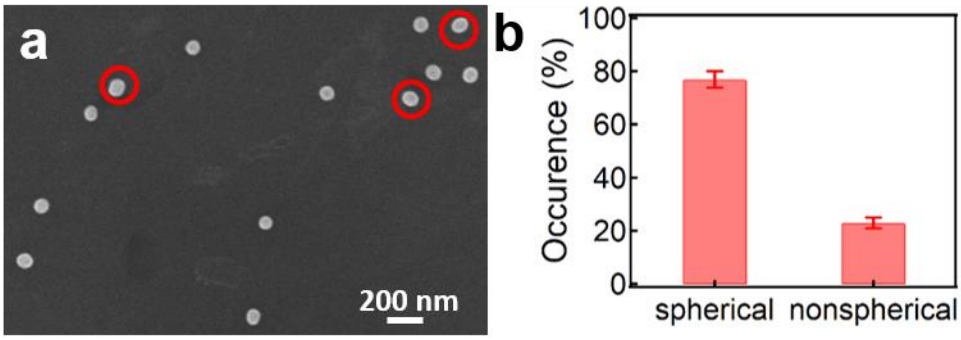


**Supplementary Fig. 34. SEM image of nonspherical Au NPs mixed in the batch of chemically synthesized 80 nm Au NPs from Nanopartz solutions.**

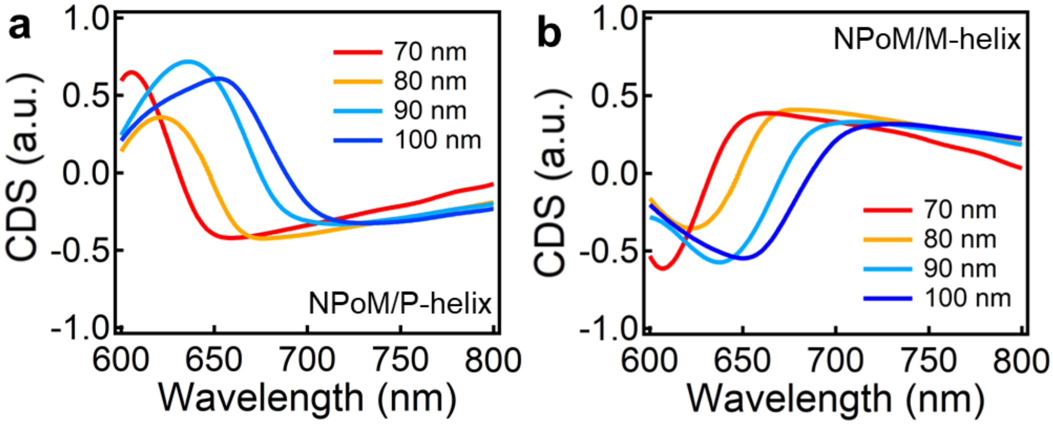


**Supplementary Fig. 35. Simulated CDS spectra of NPoM with different sizes. a** Au NPoM/P-helix and **b** Au NPoM/M-helix**.**

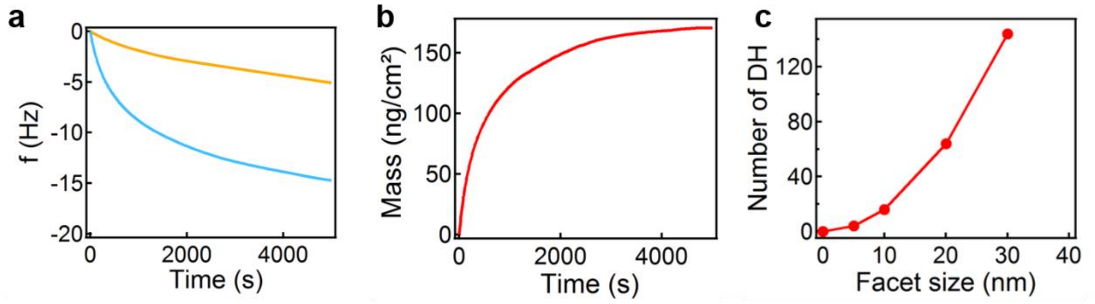


**Supplementary Fig. 36. OS Molecule density in the SAM. a** Change of quartz crystal frequency with time before (orange line) and after (blue line) absorbing OS molecules. **b** Adsorption kinetics of OS molecules on Au film. **c** Theoretical number of double helices (DH) under Au NPs of different facet sizes.

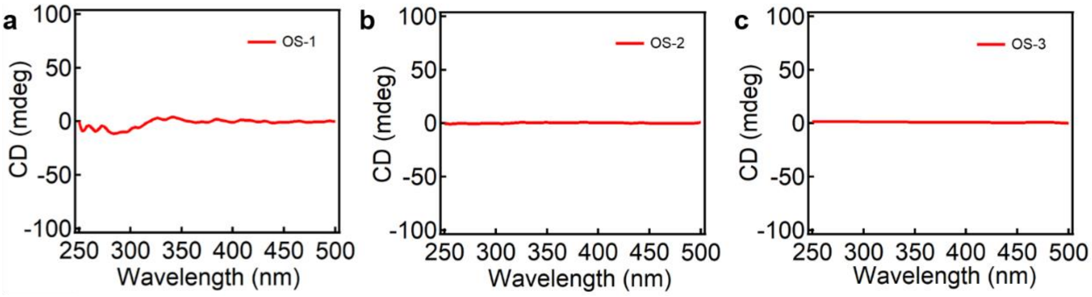


**Supplementary Fig. 37. CD spectra of racemate OS. a** OS-1, **b** OS-2. **c** OS-3.

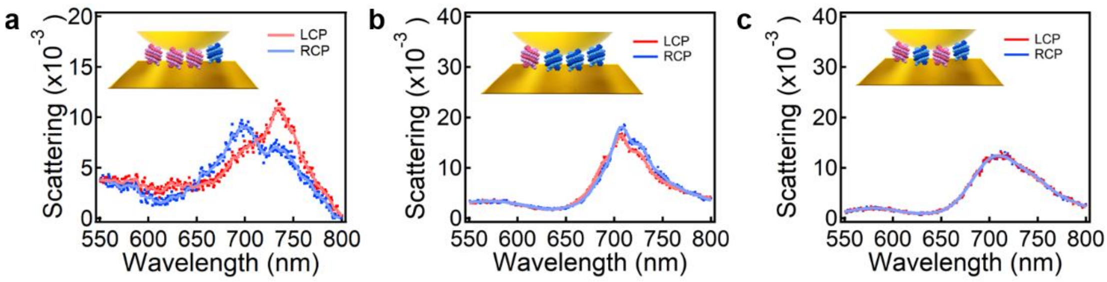


**Supplementary Fig. 38. Scattering spectra of Au NPoM with racemate of OS DHs in the nanogaps.** Schematic insets in (a-c) represent the occasions that the number of P-DHs is (a) more than (b) less than, and (c) equal to that of M-DHs, respectively.

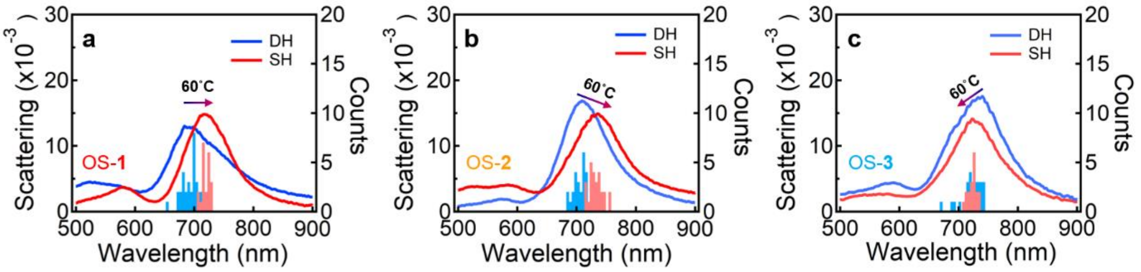


**Supplementary Fig. 39. Scattering spectra of Au NPoM/OS before and after heating, which transform from DH to SH.** (a) OS-1, (b) OS-2, (c) OS-3. The spectra are averaged based on the statistics over 30 NPoMs.

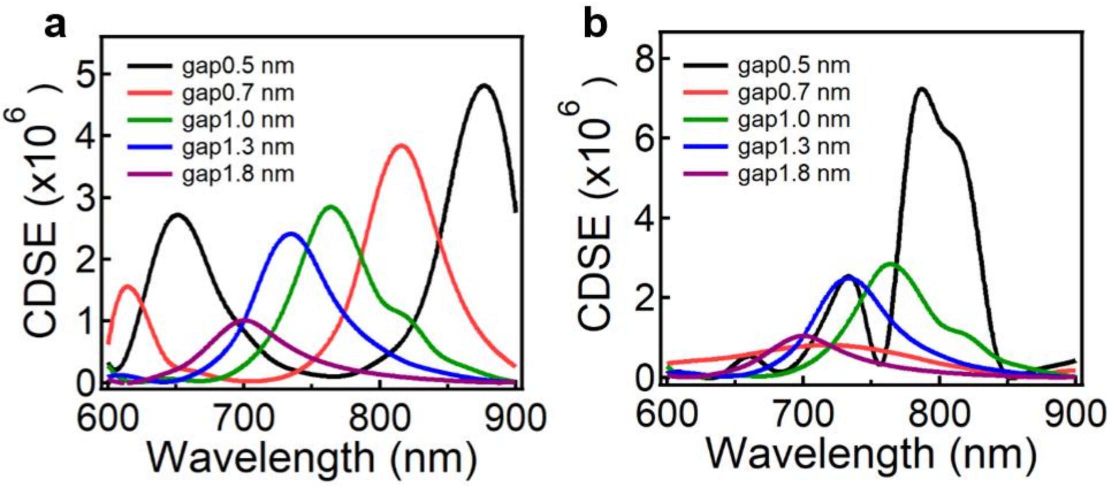


**Supplementary Fig. 40. Simulated CDSE spectra of Au/OS NPoM with varied gap sizes. a** CM model, **b** QCM model.

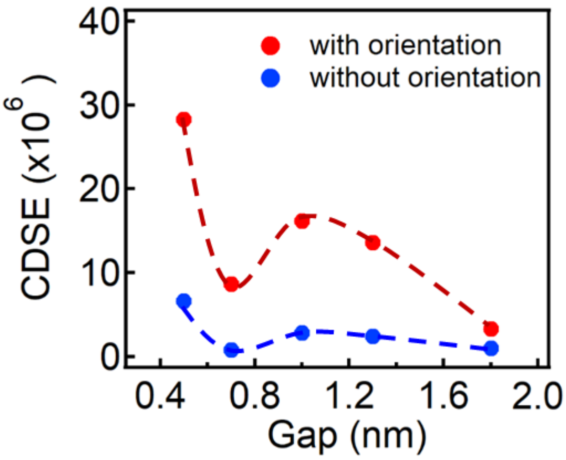


**Supplementary Fig. 41.** Comparison of CDSE calculated with and without the consideration of molecular orientation

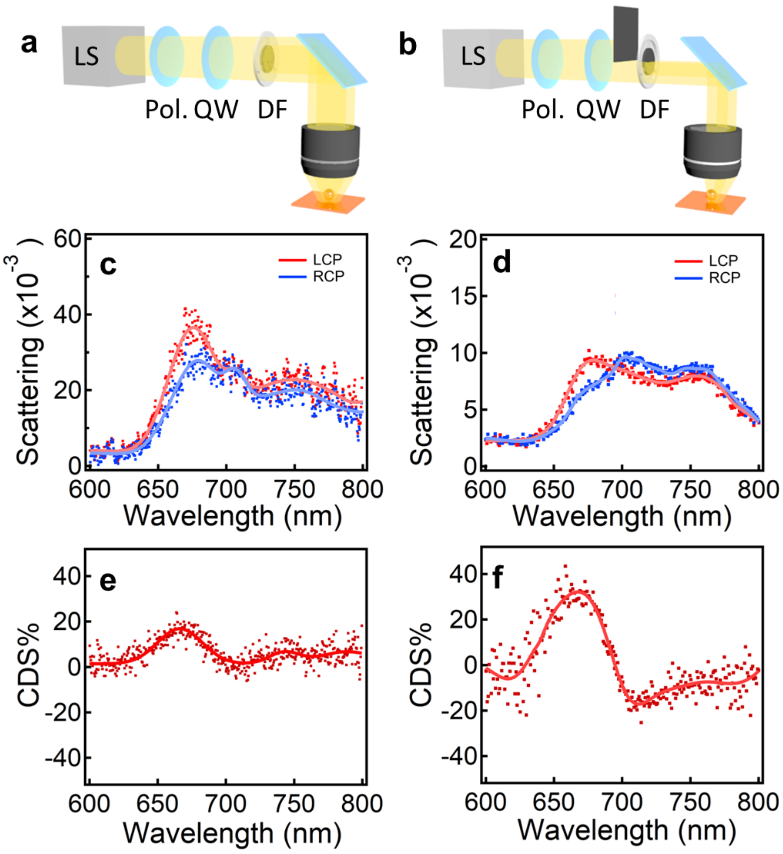


**Supplementary Fig. 42. Effect of illumination symmetry on the CDS intensity.** Schematic of **a** symmetric and **b** asymmetric DF illumination, and the resulted **c, d** scattering spectra and **e, f** CDS spectra of the same Au NPoM/OS, respectively. LS: white light source, Pol.: polarizer, QW: quarter waveplate, DF: dark field module.

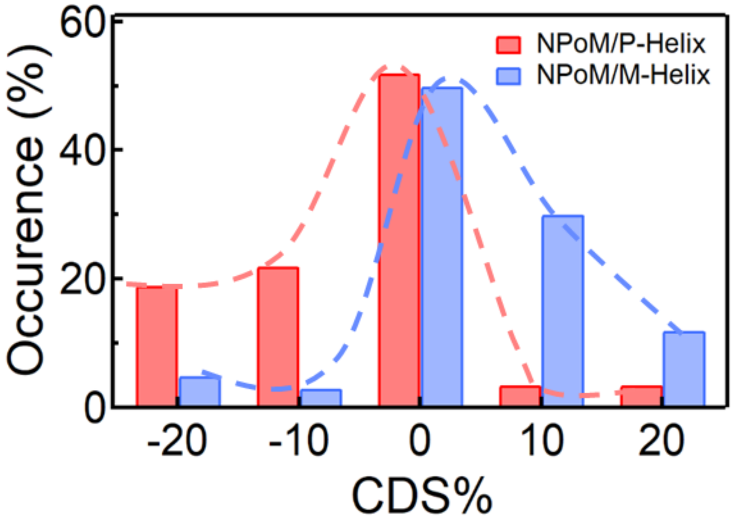


**Supplementary Fig. 43. Statistic occurrence of CDS peak intensity measured over 50 Au NPoMs with P-/M-helices in the gap using symmetric incidence as shown in Supplementary Fig. 42a.**

**Table 1. Structural summary of L-OS-1, OS-2 and OS-3.**

| | |
|---|---|
| L-OS-1 | 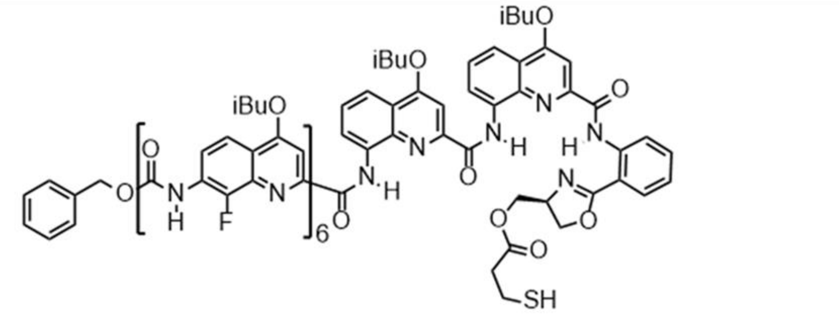 |
| OS-2 | 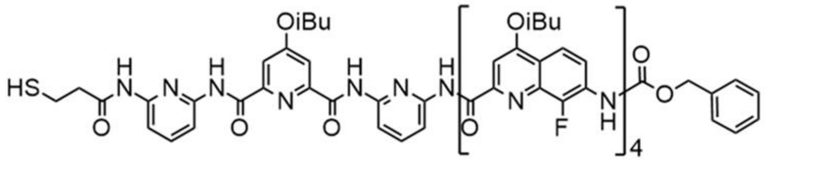 |
| OS-3 | 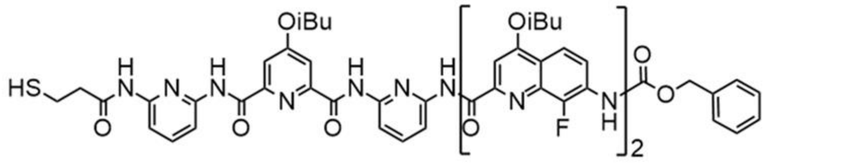 |

## Supplementary References

1. Gan Q, *et al.* Quadruple and Double Helices of 8-Fluoroquinoline Oligoamides. *Angew Chem Int Ed* **47**, 1715-1718 (2008).

2. Li D, Ma C, Xiang J, Zhang K, Yang L, Gan Q. A Disulfide Switch Providing Absolute Handedness Control in Double Helices via Conversion from the Antiparallel to Parallel Helical Pattern. *Chemistry – A European Journal* **27**, 11663-11669 (2021).
